# Chronic CD27-CD70 costimulation promotes type 1-specific polarization of effector Tregs

**DOI:** 10.3389/fimmu.2023.1023064

**Published:** 2023-03-13

**Authors:** Natalia Bowakim-Anta, Valérie Acolty, Abdulkader Azouz, Hideo Yagita, Oberdan Leo, Stanislas Goriely, Guillaume Oldenhove, Muriel Moser

**Affiliations:** ^1^Laboratory of Immunobiology, Université Libre de Bruxelles (ULB), Gosselies, Belgium; ^2^Institute for Medical Immunology, Center for Research in Immunology (U-CRI), Université Libre de Bruxelles (ULB), Gosselies, Belgium; ^3^Department of Immunology, Juntendo University School of Medicine, Tokyo, Japan

**Keywords:** CD27 costimulation, regulatory T cells, dendritic cells, eomes, CXCR3

## Abstract

**Introduction:**

Most T lymphocytes, including regulatory T cells, express the CD27 costimulatory receptor in steady state conditions. There is evidence that CD27 engagement on conventional T lymphocytes favors the development of Th1 and cytotoxic responses in mice and humans, but the impact on the regulatory lineage is unknown.

**Methods:**

In this report, we examined the effect of constitutive CD27 engagement on both regulatory and conventional CD4^+^ T cells *in vivo*, in the absence of intentional antigenic stimulation.

**Results:**

Our data show that both T cell subsets polarize into type 1 Tconvs or Tregs, characterized by cell activation, cytokine production, response to IFN-γ and CXCR3-dependent migration to inflammatory sites. Transfer experiments suggest that CD27 engagement triggers Treg activation in a cell autonomous fashion.

**Conclusion:**

We conclude that CD27 may regulate the development of Th1 immunity in peripheral tissues as well as the subsequent switch of the effector response into long-term memory.

## Graphical Abstract

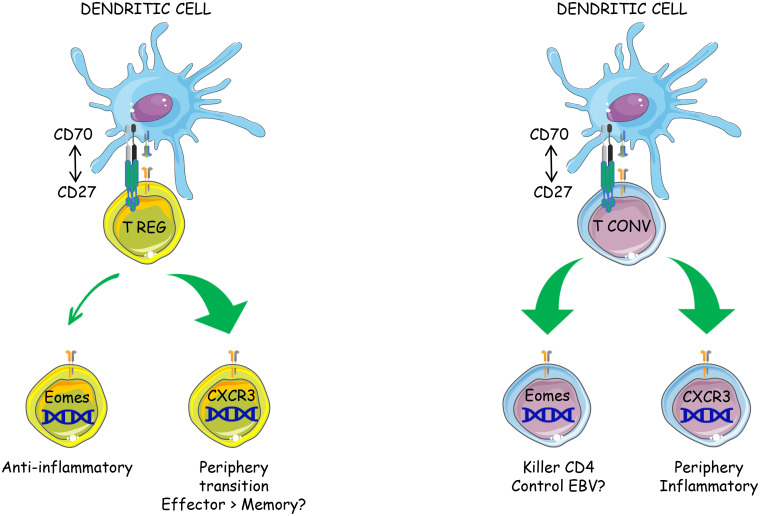

## Highlights

- CD70 overexpression by resting dendritic cells activates both Tconvs and Tregs *in vivo*
- CD27-induced Treg activation is cell autonomous
- The transcriptional program induced by CD27 engagement is similar in Tregs and Tconvs
- CD27 engagement favors the differentiation of both subsets into CXCR3^+^ type 1 effectors and the onset of a novel Eomes^+^ subpopulation

## Introduction

The discovery of costimulation in the 1990s has tremendously increased our understanding of immune regulation and opened promising avenues for immune intervention. Several costimulatory molecules from two families have been identified which may display both specific and redundant functions. The immunoglobulin superfamily comprises CD80 and CD86, discovered in 1991, as well as ICOS-L. CD80 and CD86 play a critical role in initial priming and reinforce the TCR-induced signaling pathway, leading to increased activation, IL-2 production and survival. TNF superfamily ligands include CD70, GITRL, 4-1BBL and OX40L. A recent report suggests that TNF-family ligands may be expressed at higher levels on monocyte-derived inflammatory antigen-presenting-cells (APCs), i.e. at a later stage of the response, and may therefore essentially control the post-priming accumulation/function of T lymphocytes (1). However, the intrinsic and extrinsic factors that govern the expression of the various costimulatory pathways and their respective roles in the course of immune responses remain unclear.

Among the costimulatory pathways, CD27/CD70 has gained increasing interest in 2008, when it was shown to act as a switch between immunity and tolerance *in vivo.* CD27 is constitutively expressed in most T cells, including naïve and activated CD4^+^ and CD8^+^ T cells, and provides a second signal for T cell activation (2–4). CD27 signaling is controlled by its unique ligand, CD70, which is transiently expressed upon activation on dendritic cells, B cells and T cells in a tightly regulated fashion (5–8). Keller et al. generated mice that constitutively express CD70 in conventional dendritic cells and showed that the sole expression of CD70 by immature DCs was sufficient to convert CD8^+^ T cell tolerance into immunity (9). The CD27/CD70 interaction seems to induce bidirectional intracellular signaling, with CD27 interacting with members of the TRAF family, leading to activation of NFκB and MAP kinases, and CD70 inducing PI3K and MEK activation (7, 10). There is evidence that CD27 engagement may have different outcomes, depending on the strength, duration and timing of the stimulation. The CD27/CD70 interaction has been shown to provide a potent second signal for differentiation of CD4^+^ T cells into Th1 effectors (11, 12). CD8^+^ cells into cytotoxic T lymphocytes (13–16). By contrast, persistent triggering of CD27 resulted in exhaustion and activation-induced cell death (17).

It is intriguing that regulatory T lymphocytes (Tregs) express higher levels of CD27 at steady state, as compared to conventional CD4 T cells (Tconvs), raising the question of the role of this costimulatory pathway in cell subsets displaying opposite functions. Coquet et al. demonstrated that CD27 signaling enhanced positive selection of Tregs (but not Tconvs) within the thymus, resulting in decreased Treg numbers in CD70 or CD70-deficient mice (18). Of note, Tregs have been shown to express common master transcription factors with the T helper lineage they control (19–22). We hypothesized that the CD27/CD70 pathway may regulate the activation/polarisation of both Tconvs and Tregs and more specifically the plasticity (stability versus conversion) of Tregs. To test the impact of CD27 engagement on either subset *in vivo*, we took advantage of mice that constitutively express (or not) CD70 in conventional DCs and analyzed the functional and molecular features of both populations.

## Results

### CD70 overexpression in resting DCs induces activation of Tregs and Tconvs

**Figure 1:**
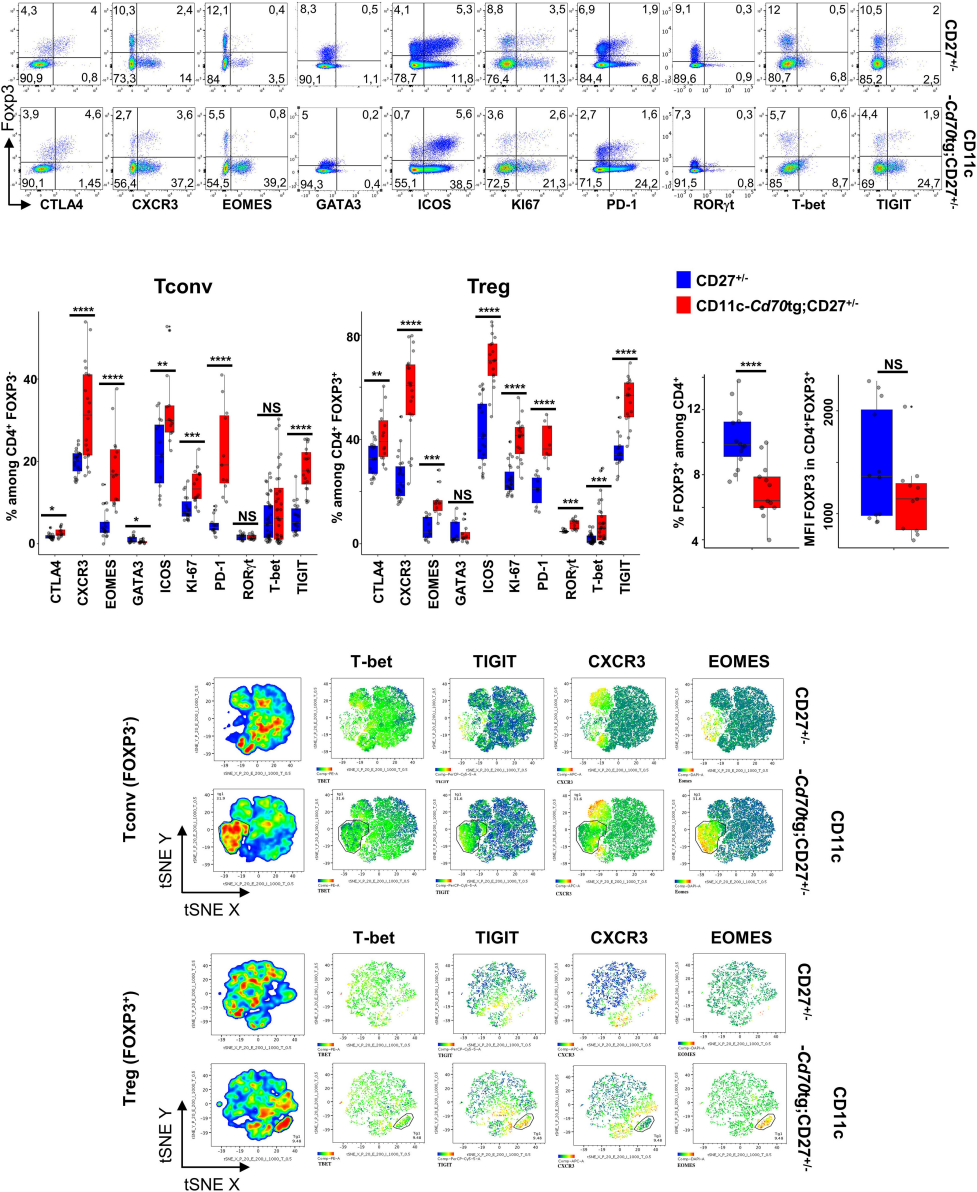
Activation of Tregs and Tconvs in CD70 transgenic mice. Spleen cells from CD27^+/-^ mice expressing or not a *CD70*tg were harvested at 6-8 wk of age and stained *ex vivo* for indicated proteins. **(A)** Spleen cells were stained for CD4, Foxp3 and the indicated markers. Singlets were selected by gating events in the diagonal of FSC-H vs FSC-A plots. Representative flow cytometry plots of indicated marker on gated viable CD4^+^ cells.**(B)** Proportion of positive cells among viable CD4^+^ Tregs and Tconvs (left panels; proportion of Tregs and intensity of Foxp3 expression (right panels). Data are from 3-4 independent experiments with 5-8 mice per group. Bars represent median ± SD. Unpaired t-test was used to determine statistical differences followed by FDR correction for multiple comparisons (*p < 0.05; **p < 0.01; ***p < 0.001; ****p < 0.0001; ns, not significant. **(C)** Representative merged (n =8) t-distributed stochastic neighbor embedding (t-SNE) plot after dimensionality reduction and unsupervised clustering of flow cytometry data from CD4^+^ Tregs and Tconvs.

To evaluate the outcome of CD27 engagement on Tconvs and Tregs, a model of supra-optimal CD27 engagement *in vivo* was devised, by crossing mice carrying a CD70 transgene under the control of the CD11c promoter (CD11c-*Cd70*tg;CD27^-/-^ mice (9, 23);) with Foxp3^eGFP^ mice (24). The heterozygous CD11c-*Cd70*tg;CD27^+/-^ mice developed a spontaneous pathology starting at about 8-9 wk of age, and died prematurely (from 13 wk of age). A phenotypic analysis of splenic CD4^+^ T lymphocytes *ex vivo* from mice of 8 weeks of age revealed an increased proliferation of both Tconvs and Tregs, with a progressive conversion into activated cells expressing Th1-type markers, i.e. Eomes (Eomesodermin), CXCR3 and TIGIT (for T cell immunoglobulin and ITIM domain) (**Figures 1A, B**). T-bet and RORγt expression was upregulated on a minor population of Tregs, Of note, the proportion of proliferating cells, which increased by 2-fold in all CD4^+^ T cells, was much higher among Tregs, reaching 41.9%, as compared to T convs (13.3%). The proportion of Eomes^+^ cells was increased by 4-fold in both subsets, reaching approximately 15.5%, whereas the expression of T-bet was increased in Tregs only (from 2.6 to 7.9%). CXCR3 was expressed by approximately 20% of lymphocytes in either cell subset in CD27^+/-^ control mice, and by 60.7% and 31.3% of Tregs and Tconvs in CD11c-CD70^tg^;CD27^+/-^ mice, respectively. The expression of ICOS was also strongly increased, reaching about 70% of Tregs. Of note, the proportion of (CD44^+^ CD62L^-^) effector Tregs was increased by 2-fold in CD70Tg mice, within a range between 20 and 35% (not depicted), whereas the proportion of Tregs expressing CXCR3 or ICOS reached 60 and 70%, respectively, indicating that the type 1 phenotype was not restricted to the effector Treg subset. The memory phenotype of Tconvs remained unchanged in the same mice. Finally, TIGIT was expressed by a much higher proportion of Tregs in control mice (34.3% versus 5.1% of Tconvs) and reached almost 60% in CD11c-*Cd70*tg;CD27^+/-^ mice, as compared to less than 20% of Tconvs. The proportion of Tregs among CD4^+^ T lymphocytes was lower in CD11c-CD70^tg^;CD27^+/-^ than in control mice, whereas the level of Foxp3 expression remained unchanged (**Figure 1B**, right panels). The distribution of Tregs remained unchanged in the liver, the mesenteric lymph nodes and the adipose tissue (**Supplemental Figure 1A**) with a notable drop in Treg frequency in the lamina propria, which correlated with intestinal dysfunction (not depicted). Note that the absolute numbers of CD4^+^ T lymphocytes were similar in both strains of mice (**Supplemental Figure 1B**).

A tSNE analysis, based on the same four markers (Eomes, T-bet, CXCR3 and TIGIT), revealed two main changes in the phenotype of CD4^+^ T lymphocytes in CD11c-*Cd70*tg;CD27^+/-^ as compared to CD27^+/-^ controls (**Figure 1C** and **Supplemental Figure 2**). First, a population of Tregs emerged (9.5% of parent), which expressed high levels of both Eomes and TIGIT, and partly CXCR3 (26,7%) and/or T-bet (5,5%). Second, the expression of CXCR3 and/or TIGIT was also increased in a significant proportion of other cells, whereas T-bet and Eomes were largely restricted to this expanded population. Similarly, a population of Tconvs (31.9% of parent) differentiated upon CD27 engagement, and displayed similar features as activated Tregs, i.e. high levels of Eomes (89.9%) and TIGIT (62.1%), and lower levels of CXCR3 (38,6%) or T-bet (3,9%). The expression of CXCR3, but not TIGIT, was also increased on a separate population of Tconvs in CD70tg mice. As expected (25), the CD27 engagement resulted in strongly reduced expression of this receptor on all T lymphocytes (not shown). Thus, the CD27 engagement without intentional TCR stimulation resulted in the expansion of a Eomes^hi^ TIGIT^hi^ cell population (representing 10% and 30% of Treg and Tconv, respectively) and increased the expression of CXCR3 on a large, distinct subpopulation in both subsets. TIGIT expression was upregulated on a large proportion of Tregs. Of note, both Tconvs and Tregs from CD11c-*Cd70*tg;CD27^+/-^ mice also expressed higher levels of co-inhibitory receptors ICOS, CTLA4 and PD-1 (**Figures 1A, B**).

**Figure 2:**
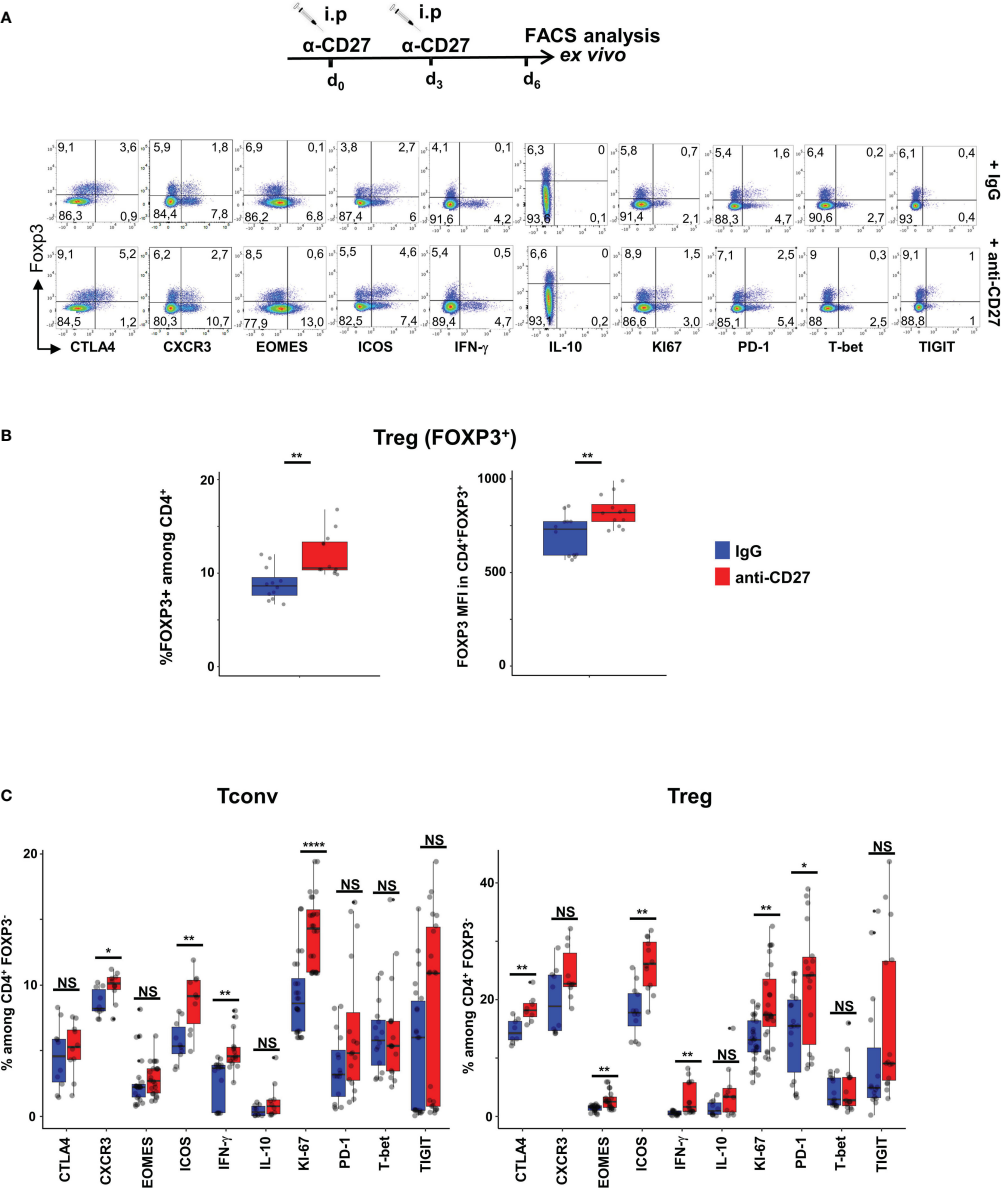
Injection of agonistic anti-CD27 mAb induces some features of Th1-type Tregs. WT mice were injected i.p. with agonistic anti-CD27 mAb (at days 0 and 3) and spleen cells were analyzed *ex vivo* by flow cytometry at day 6. **(A)** Spleen cells were stained for CD4, Foxp3 and the indicated markers? Singlets were selected by gating events in the diagonal of FSC-H vs FSC-A plots. Representative flow cytometry plots of indicated marker on gated viable CD4^+^ cells. **(B)** Proportion of Tregs and intensity of Foxp3 expression. **(C)** Proportion of CD4^+^ Tregs and Tconvs expressing the proliferation marker Ki67, transcription factors (Eomes, T-bet), cytokines (IFN-γ and IL-10) and inhibitory receptors (TIGIT, ICOS, CTLA-4 and PD-1). Data are from 3 independent experiments with 4 mice per group. Bars represent median ± SD. Unpaired t-test was used to determine statistical differences followed by FDR correction for multiple comparisons (*p < 0.05; **p < 0.01; ****p < 0.0001; ns, not significant).

To prevent an exhaustion potentially due to chronic CD70-mediated stimulation, we triggered CD27 on Tregs *in vivo* using agonistic anti-CD27 mAbs (26, 27). The data in **Figures 2A, C** indicate that Tregs from WT mice injected twice with agonistic anti-CD27 mAbs displayed partly similar phenotype changes as those observed in CD11c-*Cd70*tg;CD27^+/-^ mice; i.e. increased proliferation of Foxp3^+^ T cells, and enhanced expression of ICOS, PD-1, CTLA-4, Eomes, but not T-bet, CXCR3 nor TIGIT. Of note, the proportion of Tregs among CD4^+^ T cells increased as well as the level of Foxp3 expression (**Figure 2B**). The administration of agonistic mAbs also enhanced the proliferation of Tconvs and increased the expression of ICOS and CXCR3 on a minor population of cells. Finally, this treatment enhanced the expression of IFN-γ by both subsets. Collectively, these data indicate that CD27 may regulate the differentiation/survival of type 1 cells of regulatory and conventional lineages.

The phenotypic changes of Tregs are reminiscent of a few studies showing the development of T-bet-dependent CXCR3^+^ Treg cells in response to IFN-γ produced by effector cells (19, 28, 29). CXCR3^+^ Tregs have been shown to display unique phenotypic features and nonredundant functional properties to control Th1-related inflammation and autoimmune diseases (21, 28). We therefore sought to determine (i) the functional status of Tregs in CD11c-CD70^tg^;CD27^+/-^; (ii) the transcriptional signature of CD27 engagement *in vivo* in Tregs versus Tconvs

### Transgenic expression of CD70 on DCs induces the activation of type 1- Tconvs and Tregs

**Figure 3:**
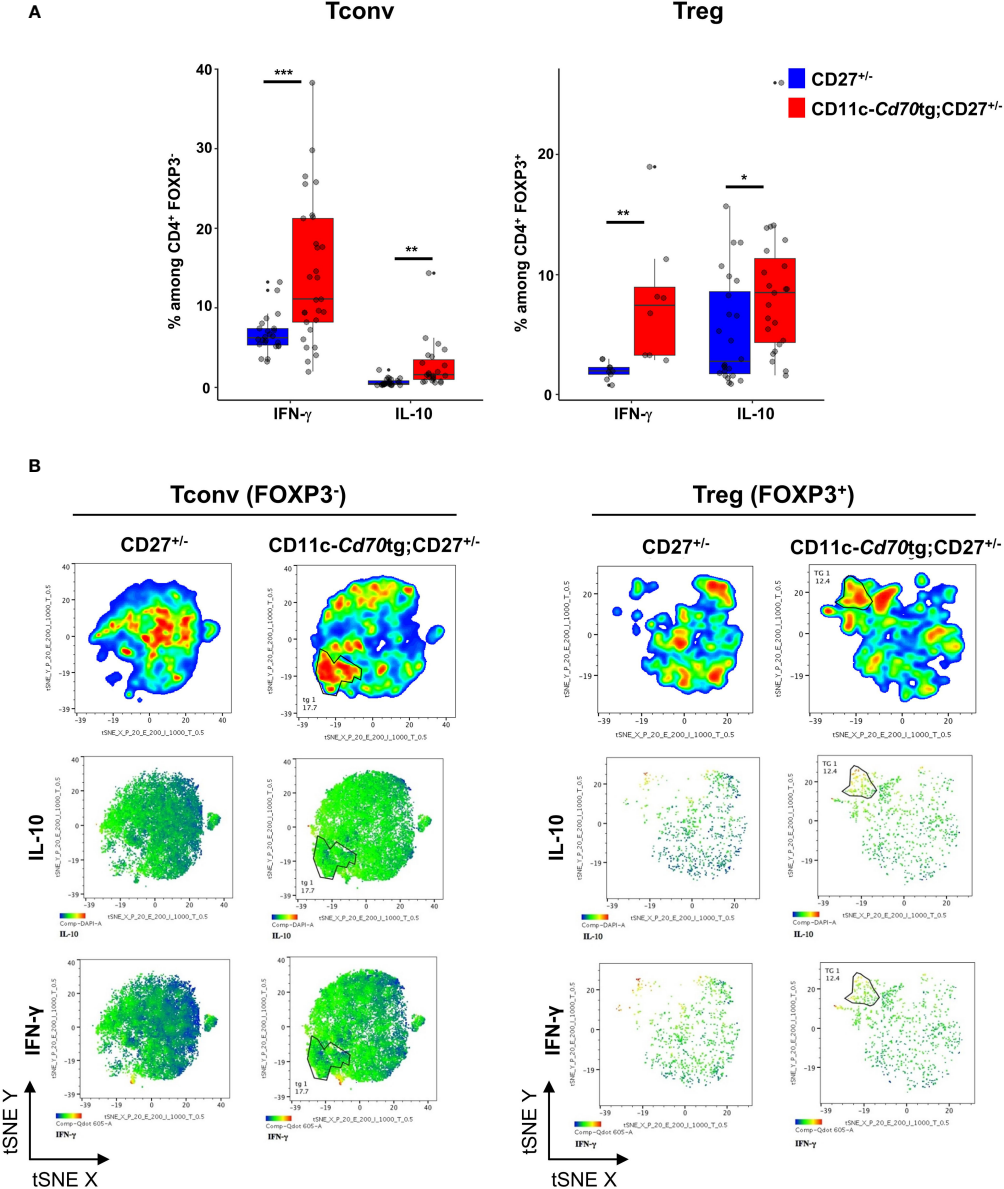
Transgenic expression of CD70 on dendritic cells induces the differentiation of type 1 effectors. **(A)** Proportion of Tregs (left) and Tconvs (right) expressing IFN-γ or IL-10 after short stimulation *in vitro* with phorbol myristate acetate (PMA)-ionomycin in the presence of brefeldin A. Data are from 3 independent experiments with 3-8 mice per group. Bars represent median ± SD. Unpaired t-test was used to determine statistical differences followed by FDR correction for multiple comparisons (*p < 0.05; **p < 0.01; ***p < 0.001; ns, not significant). **(B)** Merged (n = 8) tSNE plot after dimensionality reduction and unsupervised clustering of flow cytometry data from Tconvs and Tregs. The tSNE was built on the 3 markers and both cytokines.

We next examined the capacity of both CD4^+^ T cell subsets to express IFN-γ and IL-10. *Ex vivo* intracellular FACS staining revealed that a range of 0-40% (median: 11,1%) of Tconvs from CD11c-*Cd70*tg;CD27^+/-^ mice expressed IFN-γ in response to calcium ionophore and PMA, as compared to less than 7% (median: 6.25) from CD27^+/-^ mice (**Figure 3A**). The proportion of Tregs expressing IFN-γ *ex vivo* was also increased in mice constitutively expressing CD70, reaching about 7.45%, as compared to 2% in control mice. Both subsets displayed an increased expression of IL-10, from 2.75 to 8.5% for Tregs and 0.5 to 1.6 for Tconvs. Thus, CD27 engagement *in vivo* resulted in increased expression of IFN-γ and IL-10 in both subsets, with Tconvs and Tregs as the major IFN-γ and IL-10 producers among CD4^+^ cells, respectively. The analysis of IFN-γ and IL-10 expression with the selected combination of markers (tSNE **Figure 3B**) indicated that the majority of the IFN-γ and IL-10 producers were located in the expanded Eomes^hi^TIGIT^hi^ populations (as defined in **Figure 1C**). Thus, this subpopulation of Tregs included 36% IFN-γ^+^ and 35% IL-10^+^ cells, whereas the equivalent subset of Tconvs comprised 11,2% IFN-γ^+^ and 3.4% IL-10^+^ cells (**Supplemental Figure 3**; note that the expression of some markers was altered by the permeabilization step and PMA/calcium ionophore activation).

**Figure 4:**
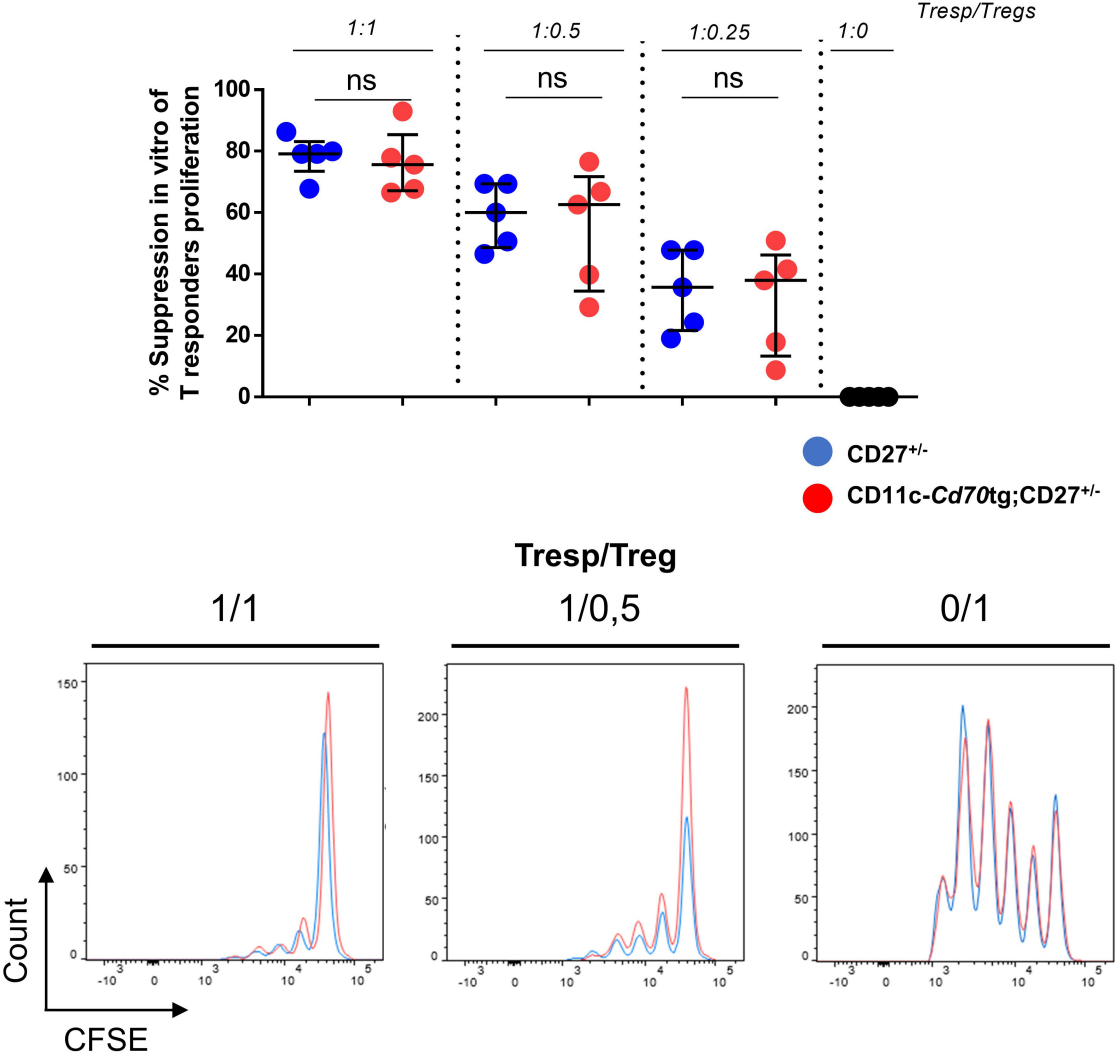
Unchanged suppressive capacity of Tregs in CD11c-*Cd70*tg;CD27^+/-^ mice. CD4^+^ Foxp3^+^ cells were sorted from CD27^+/-^ or CD11c-*Cd70*tg;CD27^+/-^ mice and co-cultured with CFSE-labeled, naive conventional CD4^+^ T cells from CD45.1 mice in the presence of soluble anti-CD3 mAb and irradiated splenocytes. After 3 days, flow cytometry profiles of CFSE were analyzed. Percent of suppression of proliferation as compared to cultures in which Treg cells were omitted. Data are representative of 4 independent experiments with n = 5 per group. Values are presented as the median ± SD and were compared by two-tailed unpaired student’s t-test. ns, not significant.

The increased expression of IFN-γ and IL-10 by CD4^+^ T lymphocytes raised the question of their functionality. To evaluate the function of Tregs, we co-cultured Tconvs from WT mice with increasing numbers of Tregs from CD27^+/-^ or CD11c-*Cd70*tg;CD27^+/-^ mice in the presence of anti-CD3 and irradiated APCs. The data in **Figure 4** suggest that CD27 engagement did not alter the suppressive capacity of Tregs, as assessed by the similar decrease in proliferation of Tconvs co-cultured with Tregs isolated from either strain.

### CD27 engagement results in Treg activation in a cell autonomous fashion

**Figure 5:**
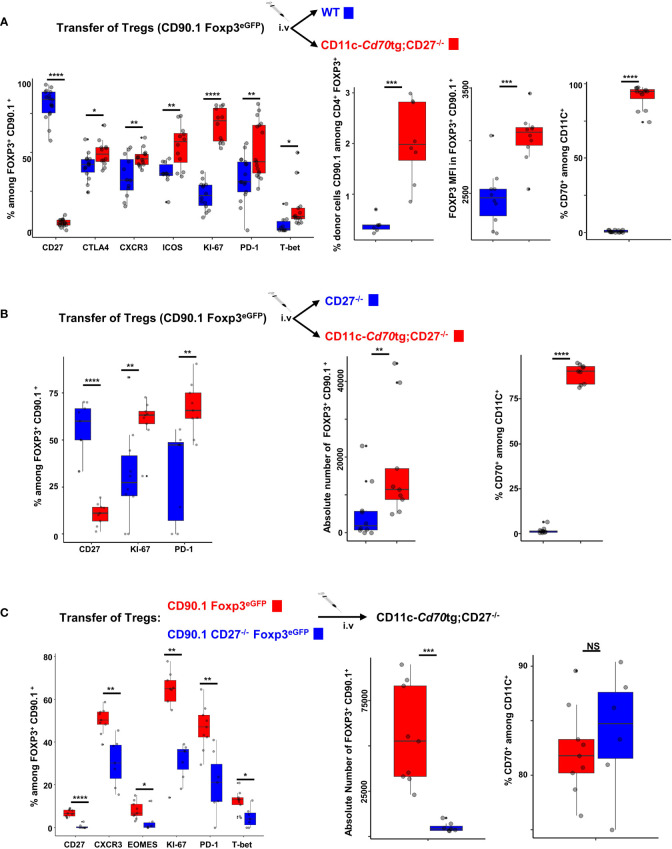
Cell autonomous activation/differentiation of Tregs upon CD27 engagement. **(A, B)** 5 x 10^5^ Tregs purified from Foxp3eGFP CD90.1 mice were injected i.v. into CD11c-*Cd70*tg;CD27^-/-^ recipients and either WT **(A)** or CD27^-/-^ **(B)** as control recipients. **(C)** Tregs were purified from Foxp3eGFP CD90.1 mice either CD27 competent or deficient (CD27^-/-^) and 5 x 10^5^ cells were injected i.v. into CD11c-*Cd70*tg;CD27^-/-^ recipients. Spleen cells were analyzed *ex vivo* by flow cytometry 7 days after injection. Data show the proportion of transferred Tregs (Foxp3^+^ CD90.1^+^) expressing the proliferation marker Ki67, transcription factors (eomes, T-bet), chemokine receptor CXCR3 and inhibitory receptors (ICOS, CTLA-4 and PD-1) as well as the absolute number of CD90.1^+^ Tregs recovered. Controls include CD27 and CD70 staining. Data are representative of 3 independent experiments with 4 mice per group. Bars represent median ± SD. Unpaired t-test was used to determine statistical differences followed by FDR correction for multiple comparisons (*p < 0.05; **p < 0.01; ***p < 0.001; ****p < 0.0001; ns, not significant).

An important question is whether the effect of CD27 engagement on Tregs is cell autonomous, or a consequence of pro-inflammatory factors produced by CD70-activated Tconvs. To address this question, we transferred purified Tregs from CD90.1 Foxp3^eGFP^ mice into CD11c-*Cd70*tg or control mice, i.e. WT (**Figure 5A**) or CD27^-/-^ (**Figure 5B**) recipients. Six days after transfer, the proportion (median) of CD90.1^+^ Tregs expressing KI67 (70.35 versus 22.1%), CXCR3 (45.9 versus 32.15%), T-bet (8.7 versus 0.8%), CTLA-4 (48.9 versus 42.3%), ICOS (56.7 versus 36.5%) and/or PD-1 (44 versus 31.1%) was increased in CD11c-*Cd70*tg;CD27^-/-^, as compared to WT hosts (**Figure 5A**). Note that the proportion of donor Tregs and their relative Foxp3 expression were much higher when transferred in recipients expressing a CD70 transgene. The CD27 expression was abrogated on transferred Tregs, a likely consequence of its engagement by its natural ligand expressed by most DCs (**Figure 5** right panels), as previously demonstrated (25). To confirm these observations, we transferred Tregs into CD27^-/-^ hosts (which display impaired Treg differentiation) expressing or not CD70tg and found similar *in vivo* cell expansion/survival and phenotypic changes, resulting in a 10-fold increase in the number of transferred Tregs when CD70tg was present (**Figure 5B**). CD27 engagement was required, as adoptive transfer of CD27-deficient Tregs into CD70tg hosts resulted in lower expression of “type 1 markers” and lower numbers of donor Tregs detected in the host, as compared to CD27-sufficient donor Tregs (**Figure 5C**). These observations indicate that CD27 engagement on Tregs was sufficient to induce their proliferation and differentiation into effectors, demonstrating an intrinsic effect.

Finally, to compare the effect of CD27 engagement on Tconvs versus Tregs, we transferred purified CD4^+^ T lymphocytes from CD90.1 Foxp3^eGFP^ mice into CD11c-*Cd70*tg, CD27^-/-^ or WT recipients. A phenotypic analysis performed 7 days after transfer (**Supplemental Figure 4**) showed a more than 2-fold increase in the proportion of Tregs among transferred cells (panel A), which correlated with higher proliferation of Tregs as compared to Tconvs (panel B). The expression of Foxp3 was increased on cells transferred into a CD70^Tg^ host, as compared to WT host (panel A). Of note, the proliferation and CXCR3 expression of transferred, but not host, cells were increased, strongly suggesting that the altered phenotype was driven by CD27-CD70 interactions.

### Common transcriptional signature of CD27 engagement in Tregs versus Tconvs

**Figure 6:**
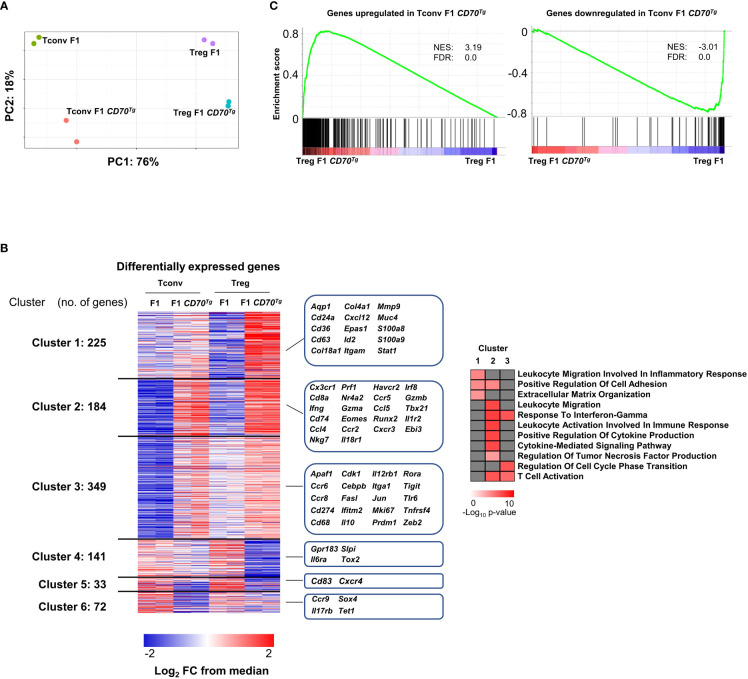
Common transcriptional signature of CD27 engagement in Tregs versus Tconvs. **(A)** Left panel: principal component analysis (PCA), a dimensionality reduction method, show the separation among Tregs and Tconvs due to CD70^Tg^. Variance in PC1 and PC2 is shown. **(B)** Left panel: differentially expressed genes of Tconvs and Tregs are clustered based on occurrence. Clusters 1 and 3 are upregulated specifically in Treg F1 CD70^Tg^ and Tconv F1 CD70^Tg^, respectively, whereas clusters 4 and 6 consist of genes downregulated specifically in Treg F1 CD70^Tg^ and Tconv F1 CD70^Tg^, respectively. Shared up/down regulated genes are found in cluster 2 and 5. Values are represented as Log2 fold-change obtained from median CPM of each gene. Selected genes for each cluster are displayed in the right margin. The number of genes in each cluster is shown in the left margin. Right panel, selected pathways enriched in clusters 1, 2, and 3 using clusterProfiler R package with default parameters and presented as −Log_10_ of p-value. **(C)** GSEA performed on F1 CD70^Tg^ data set and Tconv F1 CD70^Tg^ differentially expressed genes as gene sets. Normalized enrichment score (NES) and false discovery rate (FDR) are indicated. F1 mice: CD27^+/-^; F1 CD70^Tg^ mice: CD11c-*Cd70*tg;CD27^+/-^.

We next performed global transcriptional profiling on sorted Tregs and Tconvs (**Supplemental Figure 5**) from CD27^+/-^ mice expressing or not the CD70 transgene (**Supplemental File 2**). 471/92 and 280/92 genes were significantly up/downregulated in Tconvs and Tregs, respectively (at a Log2 fold change>1, min 60 CPM reads per gene, FdR value <0.05)(see volcano plot in **Supplemental Figure 6**). As expected, Tregs and Tconvs were clearly distinct in principal components analysis. Cells from CD11c-*Cd70*tg;CD27^+/-^ mice appeared to cluster separately from their CD27^+/-^ counterparts (**Figure 6A**). Among the differentially expressed genes, we defined 6 gene clusters based on their behaviors in both comparisons (**Figure 6B**). Among the 758 genes upregulated upon CD27 engagement in either Tconvs or Tregs, cluster 1 genes were significantly upregulated in Tregs only and encode some components of inflammation (*Stat1*, *S100a8/9*); cluster 2 includes transcripts enhanced in both Tregs and Tconvs and encodes molecules specific for inflammatory/cytotoxic responses (*Ifng, Nkg7, Prf1, Gzma/b, Cxcr3, Tbx21*, …); cluster 3 genes were preferentially upregulated in Tconvs (with a tendency in Tregs), reaching similar expression levels in both subsets, and are involved in immune regulation (*Zeb2, Prdm1; Tigit, Cebpb*). Of note, 45% of transcripts upregulated in Tregs upon CD27 engagement were similarly enriched in Tconvs. As expected (30), genes downregulated upon CD27 engagement included genes of the IL-17 pathway: *Il17rb* (in Tconvs) and *Il6ra* (in Tregs).

A gene ontology analysis (**Figure 6B**, right panel) revealed a specific enrichment of common genes involved in T cell activation and migration, production of cytokines and cytokine-mediated signaling pathway in both Tconvs and Tregs (cluster 2). The cluster 1 includes pathways upregulated mainly in Tregs, related to inflammation, cell adhesion and extracellular matrix organization, whereas the cluster 3 comprises pathways upregulated essentially in Tconvs (response to IFN-γ, regulation of cell cycle transition and T cell activation).

The overlay of the CD27-dependent Tconv signature on the comparison of CD11c-*Cd70*tg;CD27^+/-^ versus CD27^+/-^ Treg signature revealed a strong similarity between transcriptional changes in both subsets (GSEA in **Figure 6C**). These data indicate that the transcriptional program induced by CD27 engagement is similar in Tregs and Tconvs and is reminiscent of the Th1-type signature.

### Eomes overexpression and CXCR3 expression partially recapitulate CD27-induced transcriptional program in Tregs

**Figure 7:**
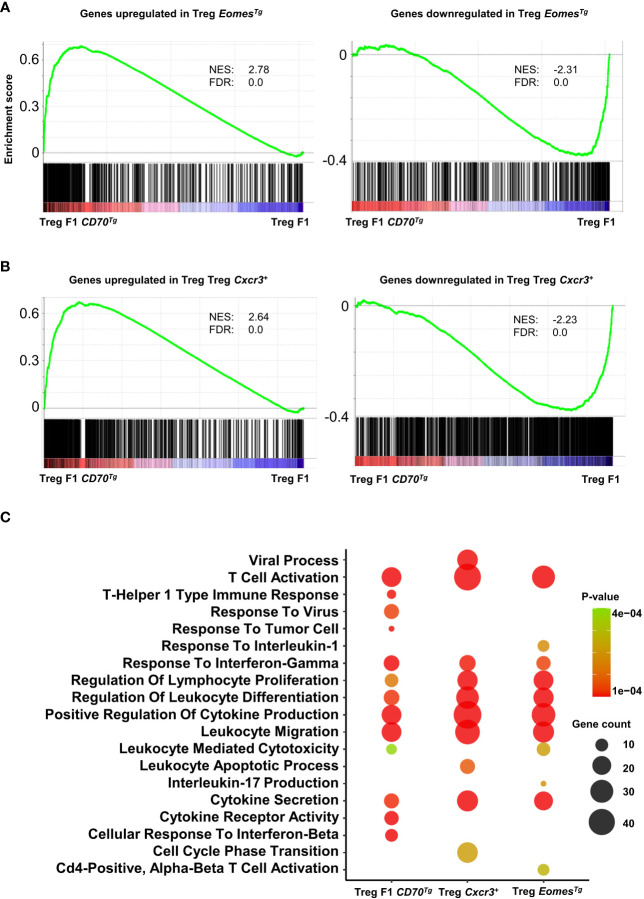
Common enriched pathways in Tregs from CD11c-*Cd70*tg;CD27^+/-^, *Eomes*^Tg^ and CXCR3^+^ Tregs. **(A, B)** GSEA performed on F1 CD70^Tg^ data set and Treg *Eomes^Tg^* **(A)** or Treg *Cxcr3^+^* **(B)** differentially expressed genes as gene sets. Normalized enrichment score (NES) and false discovery rate (FDR) are indicated. **(C)** Bubble plot for selected pathways (database MsigDB; https://www.gsea-msigdb.org/gsea/msigdb/) enriched from genes upregulated in Treg F1 *CD70*^Tg^, Treg *Cxcr3^+^* and Treg *Eomes^Tg^* using clusterProfiler R package with default parameters and presented as −Log_10_ of p-value. Color intensity indicates p-value and bubble size indicates number of enriched genes.

In order to assess the role of Eomes under steady-state conditions independently of TCR signaling, we used a transgenic mouse line that overexpresses this transcription factor in developing thymocytes under the control of the human CD2 (hCD2) regulatory elements (promoter and Locis Control Region) (31). We sorted splenic CD4^+^ Tregs from these mice and from WT controls and analyzed their transcriptome (**Supplemental File 2**). Eomes-induced and repressed genes were very highly enriched in Tregs from CD11c-*Cd70*tg;CD27^+/-^ mice, supporting the notion that this transcription factor is sufficient to drive a similar program (**Figure 7A**). A flow cytometry analysis confirmed the type 1 phenotype of Tregs from *Eomes*^Tg^, as assessed by increased expression of Tbet, CXCR3, TIGIT and PD-1, as compared to cells from WT mice (**Supplemental Figure 7**).

We next compared the signature of Tregs from CD11c-*Cd70*tg;CD27^+/-^ mice with CXCR3^+^ Tregs (22) and found strong similarities with CD27-induced changes (**Figure 7B**). Pathway analysis of signature genes from CD11c-*Cd70*tg;CD27^+/-^, *Eomes*^Tg^ and CXCR3^+^ Tregs from WT mice revealed a strong convergence among the 3 subsets (**Figure 7C**). Common enriched pathways are involved in T cell activation, response to IFN-γ, regulation of leukocyte differentiation, positive regulation of cytokine production and leukocyte migration. In addition to these common signatures, the leukocyte mediated cytotoxicity pathway was enriched only in Tregs from CD11c-*Cd70*tg;CD27^+/-^ and *Eomes*^Tg^.

Finally, we performed one experiment to evaluate the role for the CD27/Eomes axis in driving the activation of the Treg subpopulation. We injected anti-CD27 mAbs into Eomes sufficient or deficient mice. The data in **Supplemental Figure 8**, although preliminary, indicate that Eomes was dispensable for the induction of ICOS and PD-1 expression on Tregs and Tconvs in response to CD27 agonism, suggesting a functional redundancy, presumably with the related transcription factor T-bet. Additional experiments using single and double KO mice will be required to better define the respective role of these T-box transcription factors.

## Discussion

The main finding of this work is that constitutive CD27 engagement *in vivo*, in the absence of intentional TCR engagement, results in a gradual process of differentiation into type 1-Tconvs and Tregs. These data complete a previous report showing that transgenic expression of CD70 by dendritic cells is sufficient to induce spontaneous conversion of conventional T lymphocytes into effector cells (9) and indicate that Tregs undergo a similar process of differentiation into type 1 cells.

Our data reveal a dual effect of CD27 engagement on Tconvs and Tregs: (i) an increase in proliferation/survival and (ii) phenotypic and functional changes with the selective expansion of a population of Eomes^hi^ TIGIT^hi^ cells (30% of Tconvs and 10% of Tregs) that display the capacity to produce IFN-γ and/or IL-10 and the increased expression of the chemokine receptor CXCR3 on a large, separate subset (30% of Tconvs and 60% of Tregs). In the absence of deliberate antigenic stimulation, it is likely that TCR signals are provided by environmental and/or auto-antigens (9, 17).

### Effect of CD27 engagement on Tregs

The observation that highly purified CD27^+^ Tregs become activated (as assessed by proliferation of 60-70% of cells and increased expression of Tbet, CXCR3, ICOS, PD-1) upon transfer into CD70 transgenic hosts (**Figure 5**) suggests that CD27 signals autonomously on Tregs and does not solely rely on IFN-γ produced by surrounding Tconvs as previously suggested (19). Although a potential role of IFN-γ secreted by Tregs themselves cannot be excluded, as a significant proportion of Tregs from CD11c-*Cd70*tg;CD27^+/-^ mice have the capacity to express IFN-γ, our observations are consistent with a cell autonomous role of CD27 engagement on Treg differentiation. Our data further show that transient CD27 engagement induced a selective expansion of Tregs and likely increased the stability of Tregs, as assessed by higher levels of Foxp3 expression (**Figures 2** and **5A**; **Supplemental Figure 4A**), suggesting that CD27 costimulation may have a stronger impact on Tregs than on Tconvs. Finally, the lack of activation/expansion of (CD27-deficient) host T cells upon transfer of CD27-competent CD4^+^ T cells into CD11c-*Cd70*tg;CD27^-/-^ mice (**Supplemental Figure 4B**) further highlights the role of CD27 engagement, as opposed to systemic inflammation, in the activation CD4^+^ T lymphocytes.

Of note, our data show that the CD27 engagement profoundly impacts the phenotype of Tregs and induces similar transcriptomic changes as in Tconvs (see later discussion). Several reports have highlighted the role of CXCR3 in the recruitment of Tregs to local inflammatory sites. CXCR3^+^ type-1 Tregs have been shown to play a critical and non-redundant role in the control of Th1-type (auto)-immunity. T-bet- dependent CXCR3^+^ Tregs accumulated within the pancreatic islets (but not the spleen) in the NOD mouse model of type 1 diabetes, at a frequency that correlated inversely with the size of the inflammatory infiltrate (28). Mice that lack CXCR3 in Tregs specifically displayed an aggravated course of the experimental nephritis, that correlated with reduced Treg recruitment to the kidney and an overwhelming Th1 immune response (32). Elegant studies by Levine and Rudensky (21) indicated that elimination of T-bet expressing Tregs resulted in severe autoimmunity and suggested that T-bet regulated their stability and their spatial positioning with T-bet^+^ effector cells. Littringer et al. further showed that, during Th1 immune responses in mouse and human, Tregs that expressed a set of Th1-specific co-inhibitory receptors and cytotoxic molecules arose (22). The relevance of CD27 engagement on Tregs *in vivo* in more physiological conditions remains to be determined, but is suggested by a recent study demonstrating, that CD27 expression on Tregs was necessary to maintain tolerance and to suppress immune responses to tumours (33).

It is interesting that, in addition to their well-known anti-inflammatory function, Tregs may support the development of memory T cells, in particular *via* IL-10 or CTLA-4 (34–37). A recent report (37) shows that type 1-Tregs, expressing CXCR3, home in close proximity to CD8^+^ T and promote the generation of tissue-resident memory cells in multiple tissues, providing life-long immunosurveillance. Therefore, it is tempting to speculate that CD27 engagement may trigger the differentiation of type-1 Tregs involved in the transition of activated effector cells into quiescent memory cells in peripheral tissues.

Furthermore, our data indicate that chronic CD27 engagement triggers the emergence of a Eomes^+^ TIGIT^+^ Treg population that proliferated and included most of IFN-γ and IL-10 producers. TIGIT is an inhibitory receptor expressed on Tregs as well as activated and memory T cells. TIGIT has been shown to increase the stability and the immune suppressive role of Tregs (38, 39). T-bet was expressed in only 5.5% of this population, suggesting a major role of the transcription factor Eomes, a paralogue of T-bet, in inducing IFN-γ expression. Although Eomes expression was shown to limit Foxp3 induction, and thereby the suppressive function of Tregs (40), our data show that chronic CD27 engagement in CD11c-*Cd70*tg;CD27^+/-^ mice did not alter the expression of Foxp3 (**Figure 1B**) nor their suppressive capacity, whereas transient engagement enhanced its expression (**Figures 2B**, **5A** and **Supplemental Figure 4A**). The function of Eomes^+^ TIGIT^+^ Tregs remains to be determined but could be related to an increased cytotoxic mechanisms of suppression *in vivo*, as Eomes expression has been associated with enhanced cytotoxicity (41). Data in the literature show that Tregs, although dedicated to the inhibition of inflammatory responses, may produce IFN-γ. Although the consequence of this production is not entirely clear, a few reports indicate that Treg cell intrinsic IFN-γ production was required for their immunosuppressive function (42–44).

### Effect of CD27 engagement on Tconvs

The effect of CD27/CD70 interaction on the function of Tconvs is in agreement with previous studies, showing a preferential differentiation toward the Th1/cytotoxic lineages, as assessed by the expression of cytokines, transcription factors, chemokines and chemokine receptors involved in type 1 inflammation (9, 12, 18, 42–44). Of note, our data further show that a population of Eomes^+^ TIGIT^+^ Tconvs expands, representing 30% of Tconvs, and produces IFN-γ, and some IL-10. Our data are in accordance with a previous report showing that TIGIT^+^ CD4^+^ T cells exhibited defects in effector function, i.e. were poor producers of IL-2 and TNF-α, but produced high levels of IFN-γ (45). The role of the inhibitory receptor TIGIT remains undetermined but could be related to the exhaustion of CD4^+^ T cells chronically activated *via* CD27. Eomes was strongly upregulated in CD4^+^ Tconvs of CD11c-*Cd70*tg;CD27^+/-^ mice (3,2 log_2_ FC). This transcription factor, a paralogue of T-bet, appears to complement T-bet in triggering IFN-γ in T lymphocytes (46) and was involved in the differentiation of cytotoxic CD4^+^ T cells. Curran et al. (41) have shown that the activation of TNFR family receptors, in particular 4-1BB and to a lesser extent CD27, upregulated Eomes on CD4^+^ T lymphocytes. Eomes^+^ KLRG1^+^ CD4 T cells displayed cytotoxic properties and expressed Granzyme B. Similarly, Tconvs in CD11c-*Cd70*tg;CD27^+/-^ mice expressed granzyme B, natural killer cell granule protein 7 (NKG7), a regulator of granule exocytosis and a promoter of IFN-γ production in Th1 cells (47). There is some evidence that these killer CD4^+^ T cells may control the development of virus associated malignancies, an observation in line with the increased susceptibility of EBV-proliferative diseases in CD27-deficient patients (48, 49). Accordingly, CD70 has been shown to play a dominant role in both CD4 and CD8 EBV-mediated CTL generation (50).

### Pathophysiology of deregulated CD70 expression

The overt inflammation in CD11c-*Cd70*tg;CD27^+/-^ is reminiscent of similar data by Borst and colleagues who reported that mice with transgenic expression of CD70 on dendritic cells or B cells displayed a progressive pathology, i.e. a shift of Tconvs to an effector phenotype in absence of deliberate immunization and ultimately a lethal combined T and B cell immunodeficiency (9, 17, 51). These observations are intriguing, as a series of findings concur with a role for CD27 in increasing the regulatory function of Tregs. Indeed, we and others have shown that CD27 co-stimulation increases the proliferation/stability (as assessed by enhanced Foxp3 expression) of Tregs (52). In agreement with this concept, CD27 engagement led to an increased expression of inhibitory receptors that should in principle favor their suppressive capacity, accompanied by a moderate (1,5 fold) upregulation of IRF8, a transcription factor known to act as a “stabilisator” for Th1-suppressing Tregs (53). It is therefore presently unclear whether Tregs from CD11c-CD70^tg^;CD27^+/-^ contribute to the overall pathology (via the production of pro-inflammatory cytokines such as IFN-γ) or fail to adequately control the activity of CD27-activated Tconvs in these mice. Altered expression of chemokine receptors affecting the tissue sublocalisation of Tregs and/or a partial resistance of Tconvs to Tregs conferred by CD27 engagement represent possible explanations for the overt inflammation observed in CD11c-*Cd70*tg;CD27^+/-^ mice. Additional experiments will be required to better characterize the capacity of Tregs to prevent inflammatory reactions *in vivo* following chronic and acute CD27 stimulation.

### Importance of the CD27/CD70 pathway in Tregs

Costimulatory signals play an important role in Treg development and function. However, while CD28 appears to be required for both development (54) and expression of effector function in Tregs (55). CD27 engagement appears to promote the expansion, survival, stability and migration of Tregs into inflammatory sites, with little to no effect on intrinsic Treg suppressive capacity (see (56) for a review and our own observations indicating a lack of autoimmune disorder in mice selectively lacking CD27 expression in Tregs (data not shown)). The precise identification of the CD70-expressing cell population delivering these survival signals remains to be firmly established, since CD70 can be expressed by a wide variety of immune cells including APCs and activated T and B lymphocytes. The present data (using a transgenic mouse strain in which CD70 is selectively overexpressed by CD11c-positive cells) strongly suggest that dendritic cells, known to be a preferred target of Treg-mediated regulation (56, 57), represent an important source of CD27 signaling (9). Importantly, the sole expression of CD70 by immature DCs was shown to regulate immunity versus tolerance, highlighting the critical role of CD27-CD70 interactions at the interface between T cell and DC Our observations are in line with previous reports showing that the CD27/CD70 pathway triggers Treg development in thymic niches by rescuing differentiating cells from apoptosis (18), induces Treg accumulation in solid tumors in mice (58) and supports the generation of Tregs involved in the control of type 1 diabetes in NOD mice (59). However, these results differ from a previous report by Jannie Borst’s group showing that CD27 agonism did not induce Treg expansion (27) in a murine model of therapeutic vaccination to tumor. Most studies, including ours, found no difference in the *in vitro* suppressive function of Tregs from CD70-intact and deficient mice (18) or upon CD27 triggering (59).

In addition to this intrinsic effect on Tregs, we have shown previously that CD27^+^ Tregs have the capacity to inhibit the expression of CD70 on DCs, a mechanism reminiscent of the trans-endocytosis reported for CTLA-4/CD80-CD86, thereby restricting the availability of costimulatory signals in the local environment (57).

The respective roles of both costimulatory pathways on Tregs remain elusive. Some similarity includes their non redundant role in thymic development and homeostasis in the periphery. Of note, there seems to be a consensus in the literature indicating a predominant role of CD27 signaling in Treg development and survival over function, in agreement with our own observations.

## Conclusion

In conclusion, the data shown herein demonstrate that CD27 engagement favors the differentiation of CXCR3^+^ type 1-Tconvs and Tregs. Global transcriptional profiling of Tregs from Eomes Tg and CD11c-*Cd70*tg;CD27^+/-^ mice revealed a strong convergence between them as well as with CXCR3^+^ Tregs (22). However, the transcription factor Eomes appears dispensable for anti-CD27-induced Treg activation, an observation in line with reports suggesting functional redundancy and/or cooperativity between T-bet and Eomes (for review, see (60, 61)). The biological role of these type-1 Tregs *in vivo* remains to be determined but could be related to a unique, non-redundant function to control inflamed peripheral tissues and to support memory T cell differentiation in peripheral tissues. Thus, the CD27/CD70 pathway may contribute to the onset of inflammation and its resolution, i.e. the transition from effector to memory responses.

## Limitations of the study

Our observations suggest that CD27 engagement potentiates the anti-inflammatory capacity of Tregs. However, this statement is based essentially on their phenotype, gene expression and *in vitro* tests of suppressive activity. Future investigations should include an evaluation of their capacity to control inflammatory responses *in vivo* in various inflammatory models. Another approach could be based on the use of CD27^fl/fl^ mice (available in our laboratory) to selectively deplete CD27^+^ Tregs *in vivo* and assess their role in physiological and pathological immune responses. Finally, data should be interpreted with caution because the constitutive CD27-CD70 interaction affected T cell homeostasis and induced a progressive state of lethal immunodeficiency.

## Material and methods

### Mice

Wild-type C57Bl/6 mice were purchased from Envigo. CD11c-*Cd70*tg;CD27^-/-^ and CD27^-/-^ mice were kindly provided by Jannie Borst (NKI, Amsterdam) and crossed to Foxp3^eGFP^ mice from Alexander Rudensky (Memorial Sloan-Kettering Cancer Center), kindly provided by Professor Adrian Liston, to generate CD11c-*Cd70*tg;CD27^+/-^ Foxp3 reporter mice and CD27^+/-^ control mice. hCD2-Eomes^tg^ mice were generated as previously described (31). The Eomes floxed mice (B6.129S1(Cg)-*Eomes^tm1.1Bflu^*/J -Strain #:017293) were crossed onto CD4^Cre^ mice, both purchased from The Jackson Laboratory.

Mice were bred and maintained in a temperature-controlled (23 °C) animal care facility with free access to food and water and used at 6 to 8 weeks of age. The experiments were carried out in accordance with the relevant European laws and institutional guidelines. All experiments were performed in compliance with the relevant laws and institutional guidelines from the Animal Care and Use Committee of the Institute for Molecular Biology and Medicine (IBMM, ULB).

### Isolation of immune cells and cell sorting

Spleens were dissected from mouse spleen using 70µm cell strainers (Falcon) and further processed under sterile conditions. Single-cell suspensions were rinsed-out with RPMI-1640 (Lonza) supplemented with 10% (vol/vol) fetal calf serum (FCS), 2 mM L-glutamine, 1 mM sodium pyruvate, 0.1 mM non-essential amino acids, 40 mM β-mercaptoethanol, 100 U ml−1of penicillin, and 100 U ml−1of streptomycin (all reagents from Lonza).

CD4^+^ cells were purified by negative selection with magnetic depletion of B cells, macrophages, DCs, NK cells, granulocytes and CD8^+^ cells using a cocktail of biotinylated antibodies (anti-CD49b, DX5, eBiosciences; anti-GR1, RB6-8C5, produced in house; anti-Ter119, BioXCell; anti-CD11c, N418, produced in house; anti-CD19, BioXCell; anti-CD8β, H35, produced in house; anti-CD25, PC61.5, eBiosciences (used for Tconv-but not Treg- purification); anti-MHCII, M5/114.15.2, eBiosciences). Cells were recuperated after flow-through the magnetic column, with previous incubation with anti-biotin Microbeads (Miltenyi Biotec). Untouched cells were stained to exclude dead cells and incubated with Fc receptor-blocking antibodies CD16/32 (Fc block; BD Pharmingen) and surface staining antibody CD3^+^ and CD4^+^. Tconvs and Tregs were identified in FSC/SSC-low-to-moderate and sorted as GFP^-^ or GFP^+^ respectively using a BD FACSAria III.

### Flow cytometry

Single-cell suspensions were stained to exclude dead cells with live/dead fixable violet dead cell stain kit (Life technologies), incubated with Fc receptor-blocking antibodies CD16/32 (Fc block; BD Pharmingen), to block non-specific binding, followed by standard surface staining with fluorochrome-conjugated antibodies listed in **Table 1**. For intracellular staining, cells were fixed and permeabilized for 25 min with eBioscience™Foxp3/Transcription Factor Staining Buffer Set, Life Technologies) before intranuclear/intracytoplasmic staining.

**Table 1:** List of reagents and resources.

REAGENT or RESOURCE	SOURCE	IDENTIFIER
Antibodies		
Agonistic anti-CD27mAb (Rat anti-mouse RM7-3E5)	(Sakanishi &Yagita, 2010)	N/A
CD4 Alexa Fluor 700 (Rat anti-mouse RM4-5**)**	BD Biosciences	Cat #:557956
CD4 Pacific Blue (Rat anti-mouse(RM4-5)	BD Biosciences	Cat #: 558107
CD27 Pe-Cyanine7 (Rat anti-mouse LG7F9)	Thermo Fisher	Cat #: 25-0271-82
CD70 PerCP-eFluor710 (Rat anti-mouse FR70)	Thermo Fisher	Cat #:46-0701-82
CD90.1 PerCP-Cyanin5.5 (Mouse anti-mouse OX-7)	Biolegend	Cat #: 202516
CD152 APC (Rat anti-mouse UC10-489)	Thermo Fisher	Cat #: 17-1522-8
CD183 APC (Hamster anti-mouse CXCR3-183)	BD Biosciences	Cat #: 562266
CD278 PE (Armenian Hamster anti-mouse C398.4A)	BD Biosciences	Cat #: 565669
CD279 APC (Hamster anti-mouse J43)	BD Biosciences	Cat #: 562671
EOMES PE (Rat anti-mouse Dan11mag)	Thermo Fisher	Cat #: 12-4875-82
FOXP3 APC (Rat anti-mouse FJK-16s)	Thermo Fisher	Cat #: 17-5773-82
FOXP3 FITC (anti-mouse/rat FJK-16s)	Thermo Fisher	Cat #: 11-5773-82
IgG Purified Immunoglobulin	Sigma Aldrich	Cat#: I-8015
IL10 BV421 (Rat anti-mouse JES5-16E3)	BD Biosciences	Cat #: 566295
IL10 PE (Rat anti-mouse JES5-16E3)	BD Biosciences	Cat #: 554467
IFNγ APC (Rat anti-mouse XMG1.2)	BD Biosciences	Cat #: 554413
IFNγ BrillantViolet605 (Rat anti-mouse XMG1.2)	Biolegend	Cat #: 505839
Ki67 Alexa Fluor 700 (Rat anti-mouse B56)	BD Biosciences	Cat #: 561277
Tbet Pe (mouse anti-mouse/human eBio4B10)	Thermo Fisher	Cat #: 12-5825-82
TCRβ Chain PerCP-Cyanine 5.5 (Hamster anti-mouse) H57-597	BD Biosciences	Cat #: 560657
TIGIT PerCP-eFluor710 (Rat anti-mouse GIGD7)	Thermo Fisher	Cat #: 46-9501-80
Chemicals, Peptides, and Recombinant Proteins		
Live/dead stain	Thermo Fisher	Cat#: L34976
PMA	Sigma Aldritch	Cat#: P8139
Ionomycin	Sigma Aldritch	Cat#: I0634
Brefeldin A	Thermo Fisher	Cat#: 00-4506-51
Critical Commercial Assays		
eBioscience Fixation/Perm diluent	Thermo Fisher	Cat#:00-05223-56
eBioscience Fixation/Permeabilization concentrate	Thermo Fisher	Cat#: 00-5213-43
Permeabilization Buffer 10x	Thermo Fisher	Cat#: 00-8333-56
BD Perm/Wash	BD Biosciences	Cat#: 51-2091KZ
BD CytoFix/cytoperm	BD Biosciences	Cat#: 51-2090KZ
Experimental Models: Organisms/Strains		
Mouse: CD11c-Cd70^tg^;CD27^-/-^	(9)	N/A
Mouse: CD27^-/-^	(9)	N/A
Mouse: C57BL/6JOlaHsd	Envigo	Stock#: 5704F
Mouse: Foxp3^eGFP^	(63)	
Software and Algorithms		
FlowJo software version 9.6.4	Tree Star	N/A
GraphPad Prism 6	GraphPad software	N/A
Other		
BD FACS Aria III	BD Biosciences	N/A
BD Canto II	BD Biosciences	N/A

### Intracellular cytokine detection

For intracellular staining of IFN-γ and IL-10, cells were restimulated in triplicates in 96-well tissue culture plate for 4 h at 37°C, 5% CO2 with 50ng/ml phorbol 12-myristate 13-acetate (PMA) (Sigma) and 1μg/ml ionomycin in the presence of 3μg/ml of an inhibitor of intracellular protein transport BrefeldinA (eBioscience) prior to staining. After 4h, cells were stained for dead cells and surface markers as described previously and then fixed (PFA 2%) during 30min and permeabilized and intracellular stained in Triton 0.1% (diluted on BSA 0.5%). Cells were incubated with directly-conjugated cytokine-specific antibodies diluted in the corresponding permeabilization buffer for 30min and were washed in PBS before FACS analysis.

### *In vivo* treatment

Agonistic anti-CD27 mAb treatment: mice were injected i.p. with 100 or 200µg of agonistic anti-CD27 mAb (BioXCell BE0348), at days 0 and 3, or isotype control (BioXCell BE0089). Spleen cells were analyzed e*x vivo* by flow cytometry at day 6.

### Treg cell transfer

CD4^+^ Treg cell were enriched from the splenocyte suspension using magnetic microbeads-based CD4^+^ T cell isolation kit (MiltenyiBiotec) and MACS LS Columns (MiltenyiBiotec) according to the manufacturer’s instructions. Following separation, CD4^+^ T cells were stained with anti-CD4 Pacific Blue (BD Pharmingen). 5 x 10^5^ Tregs sorted as CD4^+^ FOXP3^+^ from FOXP3^GFP^ CD90.1 mice were injected i.v. into CD11c-CD70^tg^;CD27^-/-^, WT or CD27^-/-^ (C57BL/6) recipients. Spleen cells were analyzed by flow cytometry 7 days later.

### CFSE-proliferation suppression assay

Fresh splenic conventional T cells were sorted as CD4^+^CD25^−^ from C57BL/6 mice. Tregs were sorted from Foxp3^eGFP^ as CD4^+^CD90.1^+^GFP^+^. For carboxyfluorescein succinimidyl ester (CFSE) labelling, purified CD4^+^ T cells were resuspended in 10μg/ml of CFSE (Molecular Probes) for 10^7^ cells for 10min at 37°C in dark in RPMI-1640 0% FBS and were washed in cold RPMI-FCS 10% to neutralize CFSE action. 4×10^4^ CFSE-labeled CD4^+^CD25^−^ GFP^-^ T cells were incubated with 10^5^ irradiated splenocytes (2000 rad) with or without addition of Treg cells at the indicated ratios, and stimulated with 0.5μg/ml soluble anti-CD3 (2C11) for 72 h. Dividing cells were identified by CFSE dilution on FACS analysis.

### RNA purification and RNA sequencing

We extracted RNA from 2x10^5^ sorted CD4^+^ Foxp3^-^ or CD4^+^ Foxp3^+^ populations in CD11c-*Cd70*tg;CD27^+/-^ Foxp3 reporter mice and CD27^+/-^ mice (in duplicates), and RNA from 2x10^5^ sorted CD4^+^ CD25^+^ in hCD2-Eomes^tg^ mice and wild-type C57Bl/6 mice (in triplicate) using RNeasy Plus Mini kit according to manufacturer’sinstructions (Qiagen), and sample quality was tested on a 2100 Bioanalyzer (Agilent).

Libraries were prepared using Ovation SoLo RNA-Seq System (NuGEN Technologies) and underwent paired-end sequencing (25 × 10^6^ paired-end reads/sample, NovaSeq 6000 platform) performed by BRIGHTcore ULB-VUB, Belgium (http://www.brightcore.be). Adapters were removed with Trimmomatic-0.36. Reads were mapped to the reference genome mm10 using STAR_2.5.3 software with default parameters and sorted according to chromosome positions and indexed the resulting BAM files. Read counts were obtained using HTSeq-0.9.1. Genes with no raw read count, less than or equal to 10 in at least 1 sample were filtered out with an R script. Raw read counts were normalized, and a differential expression analysis was performed with DESeq2 by applying an adjusted *P* < 0.05 and an absolute log_2_ ratio larger than 1.

#### Data availability

RNA-Seq data that support the findings reported in this study have been deposited in the GEO Repository with the accession code no. GSE214395.

### Gene ontology analysis

We introduced gene lists resulting from differential analysis between different groups to clusterProfiler v3.16 R package (62). We used the comparison function to compare gene lists and determined any kind of gene-ontology association.

### Statistical analysis

Statistical analyses were performed using Prism6 (GraphPad Software, La Jolla, CA) and R (version 4.2.0). Unpaired t-test was used to determine statistical differences followed by FDR correction for multiple comparisons

Flow cytometry t-SNE plots of **Figures 1B**, **3B** show a pool of 8 mice. Other data are shown as boxplot displaying the distribution of data (the minimum, first quartile, median, third quartile, and maximum). In **Figures 1A**, **2**, **3A**, **4**, **5**, ‘‘n’’ indicates the number of mice per group. For each figure, the number of experiments performed is indicated. A p-value ≤ 0.05 was considered significant and is denoted in figures as follows: ∗, p < 0.05; ∗∗, p < 0.01; ∗∗∗, p < 0.001. No animal or sample was excluded from the analysis.

## Data availability statement

The data presented in the study are deposited in the GEO repository, accession number GSE214395.

## Ethics statement

The animal study was reviewed and approved by Animal Care and Use Committee of the Institute for Molecular Biology and Medicine (IBMM, ULB).

## Author contributions

NB-A, VA, AA, GO, OL, SG and MM designed the study and analyzed the data. NB-A and VA performed most experiments with the help of AA. HY provided reagents. OL, SG, GO and MM supervised the study. MM wrote the manuscript with the help of OL, SG and GO. All authors contributed to the article and approved the submitted version.

## References

[1] Chang YH Wang KC Chu KL Clouthier DL Tran AT Torres Perez MS. Dichotomous expression of TNF superfamily ligands on antigen-presenting cells controls post-priming anti-viral CD4+ T cell immunity. Immunity (2017) 47(5):943–958.e9. doi: 10.1016/j.immuni.2017.10.014 29150240

[2] Hintzen RQ Lens SM Lammers K Kuiper H Beckmann MP van Lier RA. Engagement of CD27 with its ligand CD70 provides a second signal for T cell activation. J Immunol (1995) 154(6):2612–23. doi: 10.4049/jimmunol.154.6.2612 7876536

[3] Hendriks J Xiao Y Borst J. CD27 promotes survival of activated T cells and complements CD28 in generation and establishment of the effector T cell pool. J Exp Med (2003) 198(9):1369–80. doi: 10.1084/jem.20030916 PMC2194245 14581610

[4] Yamada A Salama AD Sho M Najafian N Ito T Forman JP. CD70 signaling is critical for CD28-independent CD8 + T cell-mediated alloimmune responses *In vivo* . J Immunol (2005) 174(3):1357–64. doi: 10.4049/jimmunol.174.3.1357 15661893

[5] Tesselaar K Gravestein LA Van Schijndel GMW Borst J Van Lier RAW. Characterization of murine CD70, the ligand of the TNF receptor family member CD27. J Immunol (1997) 159(10):4959–65. doi: 10.4049/jimmunol.159.10.4959 9366422

[6] Tesselaar K Xiao Y Arens R van Schijndel GM Schuurhuis DH Mebius RE. Expression of the murine CD27 ligand CD70 *in vitro* and in vivo. JImmunol (2003) 170(1):33–40. doi: 10.4049/jimmunol.170.1.33 12496380

[7] Arens R Nolte MA Tesselaar K Heemskerk B Reedquist KA van Lier RAW. Signaling through CD70 regulates b cell activation and IgG production. J Immunol (2004) 173(6):3901–8. doi: 10.4049/jimmunol.173.6.3901 15356138

[8] Sanchez PJ McWilliams JA Haluszczak C Yagita H Kedl RM. Combined TLR/CD40 stimulation mediates potent cellular immunity by regulating dendritic cell expression of CD70 in vivo. J Immunol [Internet] (2007) 178(3):1564–72. doi: 10.4049/jimmunol.178.3.1564 17237405

[9] Keller AM Schildknecht A Xiao Y van den Broek M Borst J. Expression of costimulatory ligand CD70 on steady-state dendritic cells breaks CD8+ T cell tolerance and permits effective immunity. Immunity (2008) 29(6):934–46. doi: 10.1016/j.immuni.2008.10.009 19062317

[10] Akiba H Nakano H Nishinaka S Shindo M Kobata T Atsuta M. CD27, a member of the tumor necrosis factor receptor superfamily, activates NF-kappaB and stress-activated protein kinase/c-jun n-terminal kinase *via* TRAF2, TRAF5, and NF-kappaB-inducing kinase. J Biol Chem (1998) 273(21):13353–8. doi: 10.1074/jbc.273.21.13353 9582383

[11] Soares H Waechter H Glaichenhaus N Mougneau E Yagita H Mizenina O. A subset of dendritic cells induces CD4+ T cells to produce IFN-gamma by an IL-12-independent but CD70-dependent mechanism in vivo. J Exp Med (2007) 204(5):1095–106. doi: 10.1084/jem.20070176 PMC2118574 17438065

[12] van Oosterwijk MF Juwana H Arens R Tesselaar K van Oers MHJ Eldering E. CD27-CD70 interactions sensitise naive CD4+ T cells for IL-12-induced Th1 cell development. Int Immunol (2007) 19(6):713–8. doi: 10.1093/intimm/dxm033 17548342

[13] Peperzak V Veraar EAM Keller AM Xiao Y Borst J. The pim kinase pathway contributes to survival signaling in primed CD8+ T cells upon CD27 costimulation. J Immunol (2010) 185(11):6670–8. doi: 10.4049/jimmunol.1000159 21048108

[14] Van Gisbergen KPJM Klarenbeek PL Kragten NAM Unger P-PA Nieuwenhuis MBB Wensveen FM. The costimulatory molecule CD27 maintains clonally diverse CD8 + T cell responses of low antigen affinity to protect against viral variants. Immunity (2011) 35:97–108. doi: 10.1016/j.immuni.2011.04.020 21763160

[15] Feau S Garcia Z Arens R Yagita H Borst J Schoenberger and SP. The CD4 + T-cell help signal is transmitted from APC to CD8 + T- cells *via* CD27–CD70 interactions. J Immunother. (2012) 35(9):651–60. doi: 10.1038/ncomms1948 PMC3606886 22781761

[16] Taraban VY Rowley TF Kerr JP Buchan SL. CD27 costimulation contributes substantially to the expansion of functional memory CD8 + T cells after peptide immunization. Eur J Immunol (2013) 43(12):3314–23. doi: 10.1002/eji.201343579 24002868

[17] Tesselaar K Arens R van Schijndel GMW Baars PA van der Valk MA Borst J. Lethal T cell immunodeficiency induced by chronic costimulation *via* CD27-CD70 interactions. Nat Immunol (2003) 4(1):49–54. doi: 10.1038/ni869 12469117

[18] Coquet JM Ribot JC Bąbała N Middendorp S van der Horst G Xiao Y. Epithelial and dendritic cells in the thymic medulla promote CD4+Foxp3+ regulatory T cell development *via* the CD27-CD70 pathway. J Exp Med (2013) 210(4):715–28. doi: 10.1084/jem.20112061 PMC3620350 23547099

[19] Koch MA Tucker-Heard G Perdue NR Killebrew JR Urdahl KB Campbell DJ. The transcription factor T-bet controls regulatory T cell homeostasis and function during type 1 inflammation. Nat Immunol (2009) 10(6):595–602. doi: 10.1038/ni.1731 19412181 PMC2712126

[20] Kim B Lu H Ichiyama K Jin W Chang SH Dong C. Generation of ROR g t + antigen-specific T regulatory 17 cells from Foxp3 + precursors in autoimmunity. CellReports (2017) 21(1):195–207. doi: 10.1016/j.celrep.2017.09.021 PMC5716359 28978473

[21] Levine AG Medoza A Hemmers S Moltedo B Niec RE Schizas M. Stability and function of regulatory T cells expressing the transcription factor T-bet. Nature (2017) 546(7658):421–5. doi: 10.1038/nature22360 PMC5482236 28607488

[22] Littringer K Moresi C Rakebrandt N Zhou X Schorer M Dolowschiak T. Common features of regulatory T cell specialization during Th1 responses. Front Immunol (2018) 9(JUN):1–15. doi: 10.3389/fimmu.2018.01344 29951069 PMC6008317

[23] Brocker T Riedinger M Karjalainen K. Targeted expression of major histocompatibility complex (MHC) class II molecules demonstrates that dendritic cells can induce negative but not positive selection of thymocytes in vivo. J Exp Med (1997) 185(3):541–50. doi: 10.1084/jem.185.3.541 PMC2196043 9053454

[24] Rubtsov YP Niec R Josefowicz S Li L Darce J Benoist C. Stability of the regulatory T cell lineage in vivo. Sci (80- ) (2010) 329(5999):1667–71. doi: 10.1126/science.1191996 PMC4262151 20929851

[25] Burchill MA Tamburini BA Kedl RM. T Cells compete by cleaving cell surface CD27 and blocking access to CD70 bearing APCs. Eur J Immunol (2015) 45(11):3140–9. doi: 10.1002/eji.201545749 PMC4674305 26179759

[26] Sakanishi T Yagita H. Anti-tumor effects of depleting and non-depleting anti-CD27 monoclonal antibodies in immune-competent mice. Biochem Biophys Res Commun (2010) 393(4):829–35. doi: 10.1016/j.bbrc.2010.02.092 20171165

[27] Ahrends T Babała N Xiao Y Yagita H Van Eenennaam H Borst J. CD27 agonism plus PD-1 blockade recapitulates CD4+T-cell help in therapeutic anticancer vaccination. Cancer Res (2016) 76(10):2921–31. doi: 10.1158/0008-5472.CAN-15-3130 27020860

[28] Guan Tan T Mathis D Benoist C. Singular role for T-BET + CXCR3 + regulatory T cells in protection from autoimmune diabetes. PNAS (2016) 113(49):14103–8. doi: 10.1073/pnas.1616710113 PMC5150376 27872297

[29] Koch MA Thomas KR Perdue NR Smigiel KS Srivastava S Campbell DJ. T-Bet+ treg cells undergo abortive Th1 cell differentiation due to impaired expression of IL-12 receptor β2. Immunity (2012) 37(3):501–10. doi: 10.1016/j.immuni.2012.05.031 PMC3501343 22960221

[30] Coquet JM Middendorp S van der Horst G Kind J Veraar EAM Xiao Y. The CD27 and CD70 costimulatory pathway inhibits effector function of T helper 17 cells and attenuates associated autoimmunity. Immunity (2013) 38(1):53–65. doi: 10.1016/j.immuni.2012.09.009 23159439

[31] Istaces N Splittgerber M Lima Silva V Nguyen M Thomas S Le A. EOMES interacts with RUNX3 and BRG1 to promote innate memory cell formation through epigenetic reprogramming. Nat Commun (2019) 10(1):3306. doi: 10.1038/s41467-019-11233-6 31341159 PMC6656725

[32] Paust HJ Riedel JH Krebs CF Turner JE Brix SR Krohn S. CXCR3+ regulatory T cells control TH1 responses in crescentic GN. J Am Soc Nephrol. (2016) 27(7):1933–42. doi: 10.1681/ASN.2015020203 PMC4926966 26534920

[33] Muth S Klaric A Radsak M Schild H Probst HC. CD27 expression on treg cells limits immune responses against tumors. J Mol Med (2022) 100(3):439–49. doi: 10.1007/s00109-021-02116-9 PMC8843905 34423375

[34] Pace L Tempez A Arnold-Schrauf C Lemaitre F Bousso P Fetler L. Regulatory T cells increase the avidity of primary CD8+ T cell responses and promote memory. Sci (80- ). (2012) 338(6106):532–6. doi: 10.1126/science.1227049 23112334

[35] Kalia V Penny LA Yuzefpolskiy Y Baumann FM Sarkar S. Quiescence of memory CD8+ T cells is mediated by regulatory T cells through inhibitory receptor CTLA-4. Immunity (2015) 42(6):1116–29. doi: 10.1016/j.immuni.2015.05.023 26084026

[36] Laidlaw BJ Craft J Kaech SM. The multifaceted role of CD4+ T cells in the regulation of CD8+ T cell memory maturation. Nat Rev Immunol (2016) 16(2):102–11. doi: 10.1038/nri.2015.10 PMC4860014 26781939

[37] Ferreira C Barros L Baptista M Blankenhaus B Barros A Figueiredo-Campos P. Type 1 treg cells promote the generation of CD8+ tissue-resident memory T cells. Nat Immunol (2020) 21(7):766–76. doi: 10.1038/s41590-020-0674-9 32424367

[38] Chen F Xu Y Chen Y Shan S. TIGIT enhances CD4+ regulatory T-cell response and mediates immune suppression in a murine ovarian cancer model. Cancer Med (2020) 9(10):3584–91. doi: 10.1002/cam4.2976 PMC7221438 32212317

[39] Sato K Yamashita-Kanemaru Y Abe F Murata R Nakamura-Shinya Y Kanemaru K. DNAM-1 regulates Foxp3 expression in regulatory T cells by interfering with TIGIT under inflammatory conditions. Proc Natl Acad Sci U.S.A. (2021) 118(21):e2021309118. doi: 10.1073/pnas.2021309118 34011606 PMC8166105

[40] Lupar E Brack M Garnier L Laffont S Rauch KS Schachtrup K. Eomesodermin expression in CD4 ^+^ T cells restricts peripheral Foxp3 induction. J Immunol (2015) 195(10):4742–52. doi: 10.4049/jimmunol.1501159 26453746

[41] Curran MA Geiger TL Montalvo W Kim M Reiner SL Al-Shamkhani A. Systemic 4-1BB activation induces a novel T cell phenotype driven by high expression of eomesodermin. J Exp Med (2013) 210(4):743–55. doi: 10.1084/jem.20121190 PMC3620352 23547098

[42] Sawitzki B Kingsley CI Oliveira V Karim M Herber M Wood KJ. IFN-γ production by alloantigen-reactive regulatory T cells is important for their regulatory function in vivo. J Exp Med (2005) 201(12):1925–35. doi: 10.1084/jem.20050419 PMC2212028 15967822

[43] Koenecke C Lee C-W Thamm K Föhse L Schafferus M Mittrücker H-W. IFN-γ production by allogeneic Foxp3+ regulatory T cells is essential for preventing experimental graft-versus-Host disease. J Immunol (2012) 189(6):2890–6. doi: 10.4049/jimmunol.1200413 22869903

[44] Dikiy S Rudensky AY. Principles of regulatory T cell function. Immunity (2023) 56(2):240–55. doi: 10.1016/j.immuni.2023.01.004 36792571

[45] Zhang W Anyalebechi JC Ramonell KM Chen CW Xie J Liang Z. TIGIT modulates sepsis-induced immune dysregulation in mice with preexisting malignancy. JCI Insight (2021) 6(11):e139823. doi: 10.1172/jci.insight.139823 34100383 PMC8262279

[46] Pearce EL Mullen AC Martins GA Krawczyk CM Hutchins AS Zediak VP. Control of effector CD8+ T cell function by the transcription factor eomesodermin. Sci (80- ). (2003) 302(5647):1041–3. doi: 10.1126/science.1090148 14605368

[47] Ng SS De Labastida Rivera F Yan J Corvino D Das I Zhang P. The NK cell granule protein NKG7 regulates cytotoxic granule exocytosis and inflammation. Nat Immunol (2020) 21(10):1205–18. doi: 10.1038/s41590-020-0758-6 PMC7965849 32839608

[48] Abolhassani H Edwards ESJ Ikinciogullari A Jing H Borte S Buggert M. Combined immunodeficiency and Epstein-Barr virus–induced b cell malignancy in humans with inherited CD70 deficiency. J Exp Med (2017) 214(1):91–106. doi: 10.1084/jem.20160849 28011864 PMC5206499

[49] Izawa K Martin E Soudais C Bruneau J Boutboul D Rodriguez R. Inherited CD70 deficiency in humans reveals a critical role for the CD70-CD27 pathway in immunity to Epstein-Barr virus infection. J Exp Med (2017) 214(1):73–89. doi: 10.1084/jem.20160784 28011863 PMC5206497

[50] Choi IK Wang Z Ke Q Hong M Qian Y Zhao X. Signaling by the Epstein–Barr virus LMP1 protein induces potent cytotoxic CD4+ and CD8+ T cell responses. Proc Natl Acad Sci United States America. (2018) 115:E686–95. doi: 10.1073/pnas.1713607115 PMC5789919 29311309

[51] Arens R Tesselaar K Baars PA Van Schijndel GMW Hendriks J Pals ST. Constitutive CD27/CD70 interaction induces expansion of effector-type T cells and results in IFNγ-mediated b cell depletion. Immunity (2001) 15(5):801–12. doi: 10.1016/S1074-7613(01)00236-9 11728341

[52] Winkels H Meiler S Lievens D Engel D Spitz C Bürger C. CD27 co-stimulation increases the abundance of regulatory T cells and reduces atherosclerosis in hyperlipidaemic mice. Eur Heart J (2017) 38(48):3590–9. doi: 10.1093/eurheartj/ehx517 29045618

[53] Lee W Kim HS Baek SY Lee GR. Transcription factor IRF8 controls Th1-like regulatory T-cell function. Cell Mol Immunol (2016) 13(6):785–94. doi: 10.1038/cmi.2015.72 PMC5101446 26166768

[54] Tang Q Henriksen KJ Boden EK Aaron J Ye J Subudhi SK. Cutting edge: CD28 controls peripheral homeostasis of CD4 + CD25 + regulatory T cells. J Immunol (2003) 171):3348–52. doi: 10.4049/jimmunol.171.7.3348 14500627

[55] Zhang R Huynh A Whitcher G Chang JH Maltzman JS Turka LA. An obligate cell-intrinsic function for CD28 in tregs. J Clin Invest. (2013) 123(2):580–93. doi: 10.1172/JCI65013 PMC3561819 23281398

[56] Wing J. Co-Signaling molecules in neurological diseases. Adv Exp Med Biol (2019) 1189:233–65. doi: 10.1007/978-981-32-9717-3_9 31758537

[57] Dhainaut M Coquerelle C Uzureau S Denoeud J Acolty V Oldenhove G. Thymus-derived regulatory T cells restrain pro- inflammatory Th 1 responses by downregulating CD 70 on dendritic cells. EMBO J (2015) 34(10):1336–48. doi: 10.15252/embj.201490312 PMC4491995 25787857

[58] Claus C Riether C Schürch C Matter MS Hilmenyuk T Ochsenbein AF. CD27 signaling increases the frequency of regulatory T cells and promotes tumor growth. Cancer Res (2012) 72(14):3664–76. doi: 10.1158/0008-5472.CAN-11-2791 22628427

[59] Ye C Low BE Wiles MV Brusko TM Serreze DV Driver JP. CD70 inversely regulates regulatory T cells and invariant NKT cells and modulates type 1 diabetes in NOD mice. J Immunol (2020) 205(7):1763–77. doi: 10.4049/jimmunol.2000148 PMC7787366 32868408

[60] Pritchard GH Kedl RM Hunter CA. The evolving role of T-bet in resistance to infection. Nat Rev Immunol (2019) 19(6):398–410. doi: 10.1038/s41577-019-0145-4 30846856 PMC7272213

[61] Takeuchi A Saito T. CD4 CTL, a cytotoxic subset of CD4+ T cells, their differentiation and function. Front Immunol (2017) 8(FEB):1–7. doi: 10.3389/fimmu.2017.00194 28280496 PMC5321676

[62] Yu G Wang LG Han Y He QY. ClusterProfiler: An r package for comparing biological themes among gene clusters. Omi A J Integr Biol (2012) 16(5):284–7. doi: 10.1089/omi.2011.0118 PMC3339379 22455463

[63] Fontenot JD Rasmussen JP Williams LM Dooley JL Farr AG Rudensky AY. Regulatory T cell lineage specification by the forkhead transcription factor Foxp3. Immunity (2005) 22:329–41. doi: 10.1016/j.immuni.2005.01.016 15780990

