# Supplementary material for: Chronic CD27-CD70 costimulation promotes type 1-specific polarization of effector Tregs
Source: Front Immunol. 2023 Mar 13;14:1023064. doi: 10.3389/fimmu.2023.1023064 (PMC10041113; doi:10.3389/fimmu.2023.1023064)
Supplement: Supplementary file 3 [file DataSheet_3.pdf]

Supplemental figures

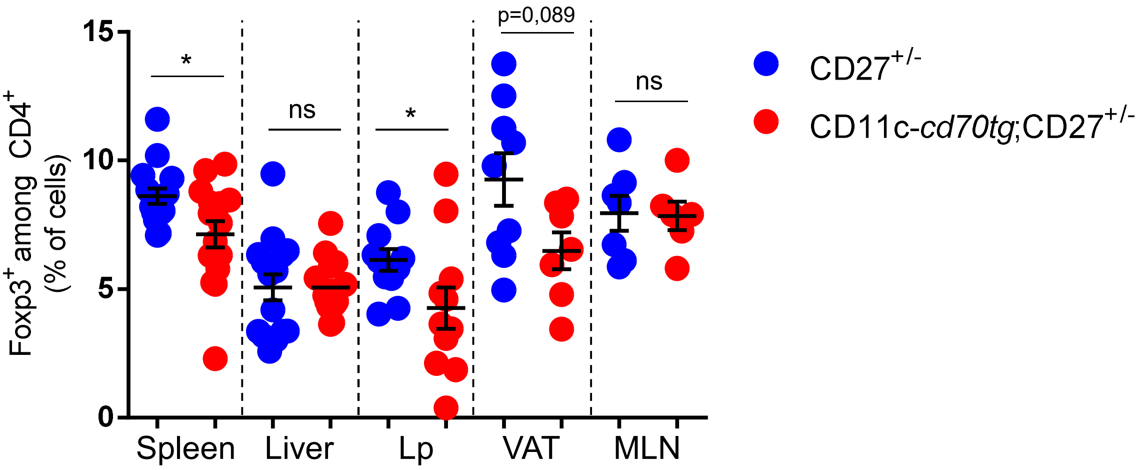

Supplemental Figure 1: Distribution of Tregs in tissues of CD27$^{+/-}$ mice expressing or not CD70tg

Spleen, liver, lamina propria (Lp), visceral adipose tissue (VAT) and mesenteric lymph nodes (MLN) were harvested from CD27$^{+/-}$ or CD11c-*cd70tg*;CD27$^{+/-}$ mice at 8 to 12 wk of age. Treg frequency of the different compartments was evaluated by flow cytometry. A non-parametric Mann-Whitney U test was used to determine statistical differences (*p<0.05; ns=not significant). Data are from 2 to 4 experiments with 2-4 mice per group.

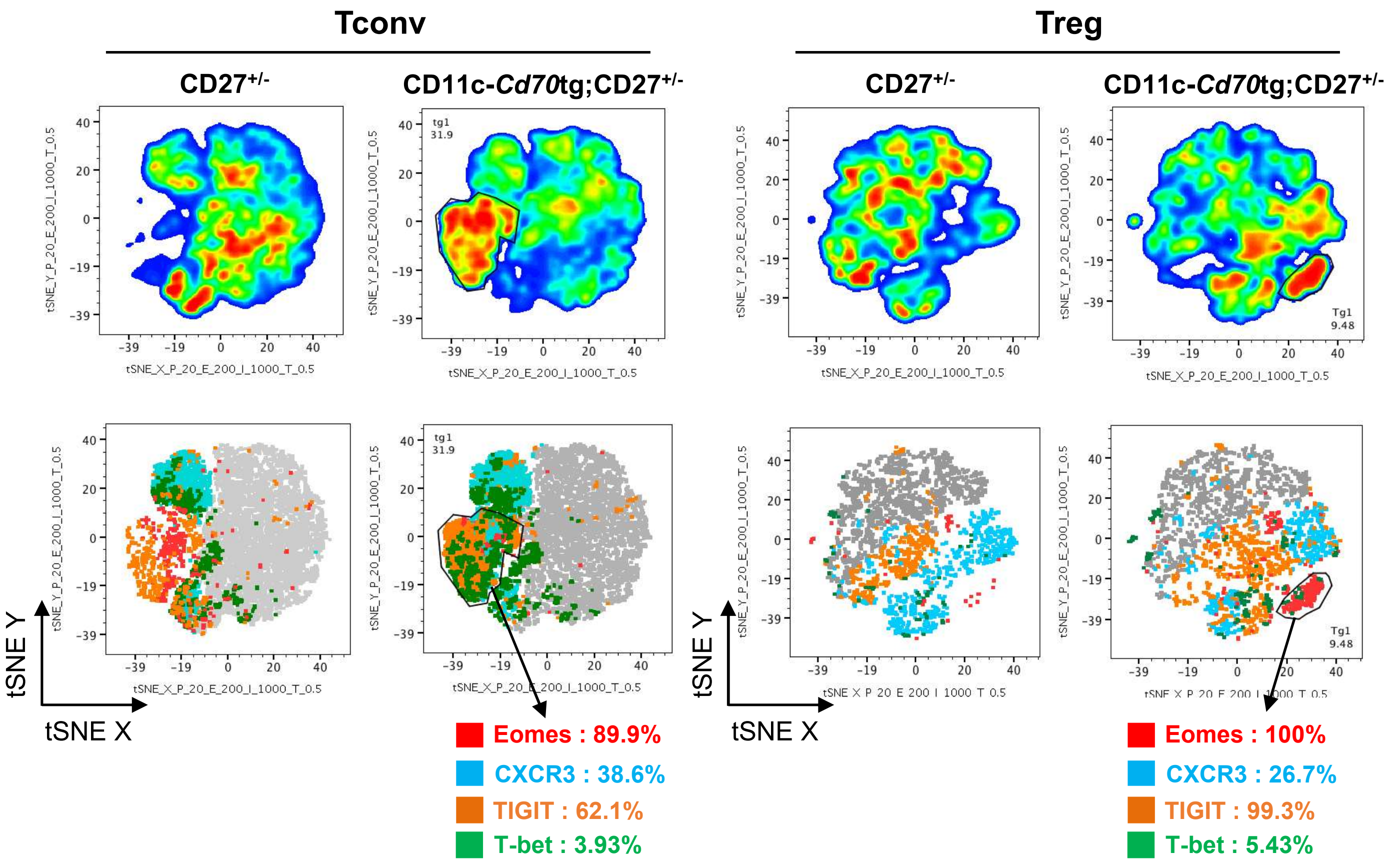

Supplemental Figure 2 : CD27 engagement induces a novel Th1-like population on Tregs and Tconvs ; related to figure 1

Spleen cells from CD27$^{+/-}$ mice expressing or not a *CD70*tg were harvested at 6-8 wk of age and stained *ex vivo* for proliferative Ki67 marker, and Th-1 like markers Eomes, Tbet, CXCR3 and Tigit expression among the new emerged population. Proportion of positive cells among viable CD4^{+} Tregs and Tconvs. Representative merged (n =8) t-distributed stochastic neighbor embedding (t-SNE) plot after dimensionality reduction and unsupervised clustering of flow cytometry data from CD4^{+} Tregs and Tconvs..

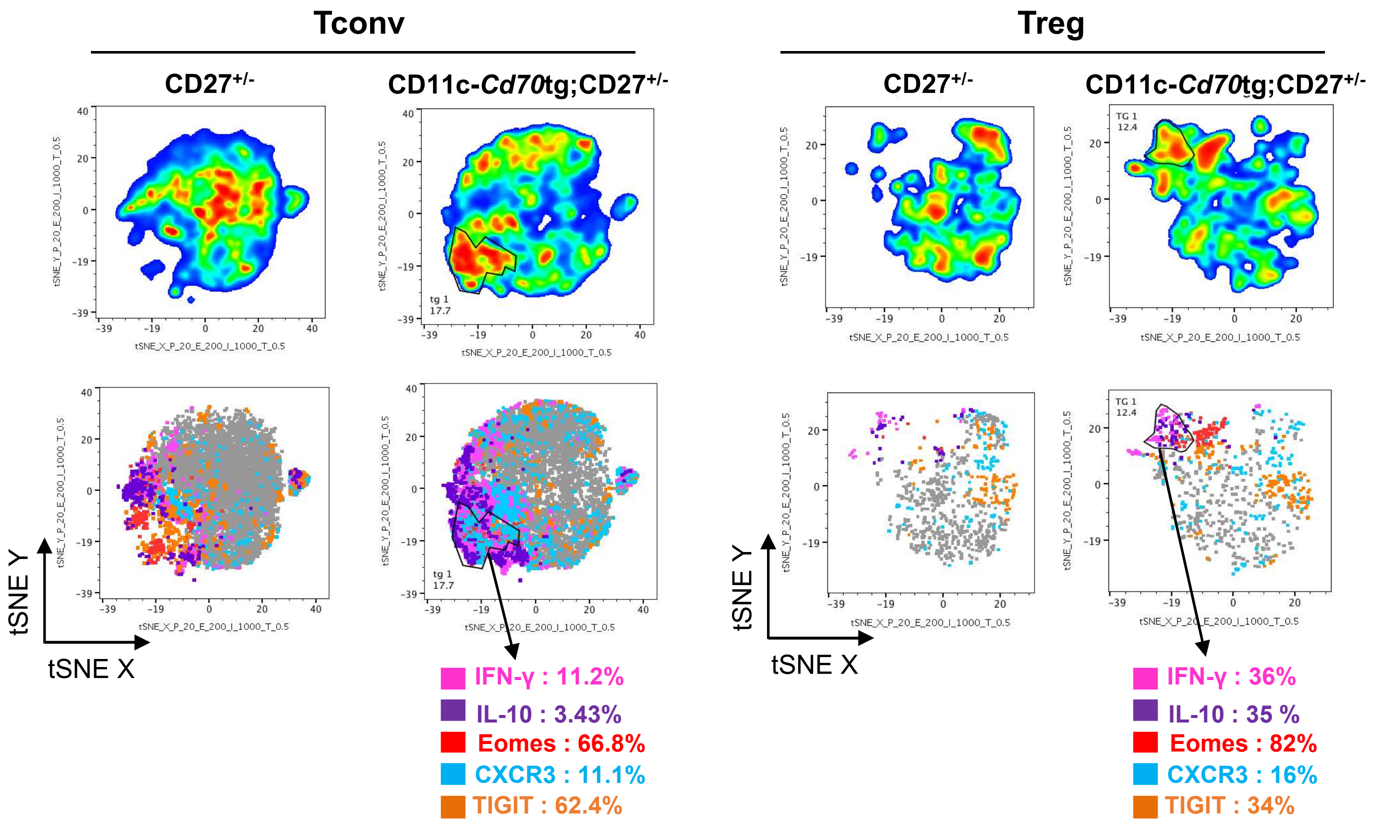

Supplemental Figure 3 : Th1-effector cytokine production among Tregs and Tconvs from CD27$^{+/-}$ and CD11c-*Cd70*tg;CD27$^{+/-}$ mice ; related to figure 1

Proportion of Tconvs (left) and Tregs (right) expressing IFN-γ or IL-10 after short stimulation *in vitro* with phorbol myristate acetate (PMA)-ionomycin in the presence of brefeldin A. Data are shown as the percentage of cells expressing cytokines/markers in the novel population in CD11c-Cd70tg;CD27+/- mice. Representative merged (n = 8) t-SNE plots after dimensionality reduction and unsupervised clustering of flow cytometry data from Tconvs and Tregs.

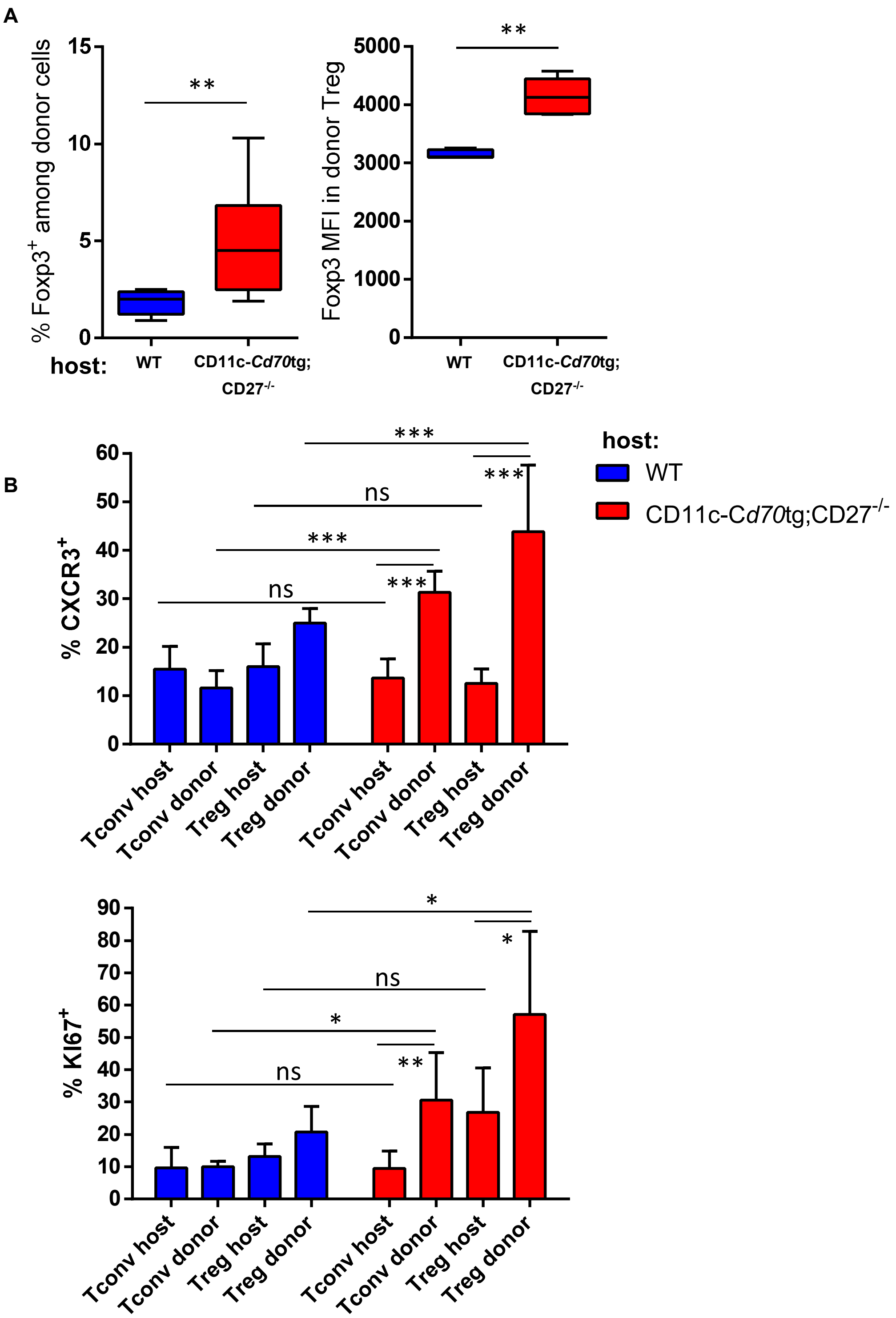

Supplemental Figure 4 : CD27 engagement is required for T cell activation

5 x 10^6 $CD4^+$ T lymphocytes purified from Foxp3eGFP CD90.1 mice were injected i.v. into CD11c-Cd70tg;$CD27^{-/-}$ or WT recipients. Spleen cells were analyzed *ex vivo* by flow cytometry 7 days after injection. Data show (A) the proportion of Tregs ($Foxp3^+$) among transferred cells ($CD90.1^+$) and the MFI of Foxp3 expression by the same cells; (B) the expression of the proliferation marker Ki67 and the chemokine receptor CXCR3 by donor and host cells. Data are representative of 2 independent experiments with 4 mice per group. A non-parametric Mann-Whitney U test was used to determine statistical differences (*p<0.05; **p<0.01; (***p<0.005, not significant).

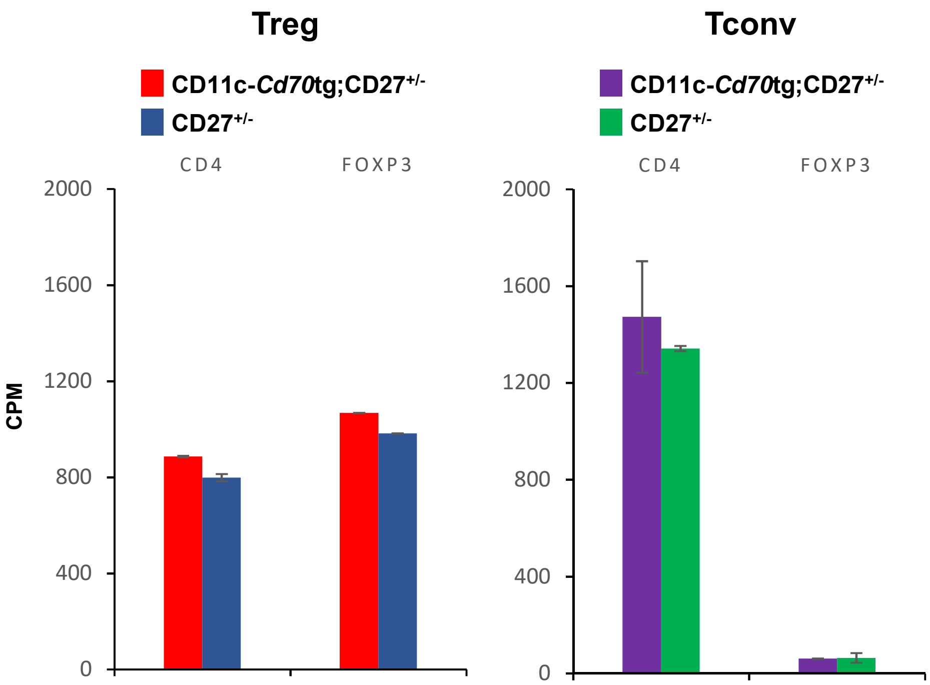

Supplemental Figure 5 : Validation of samples RNAseq ; related to figure 6

Expression of CD4 and Foxp3 mRNA by $CD4^+$ $Foxp3^-$ (Tconvs) and $Foxp3^+$ (Tregs)

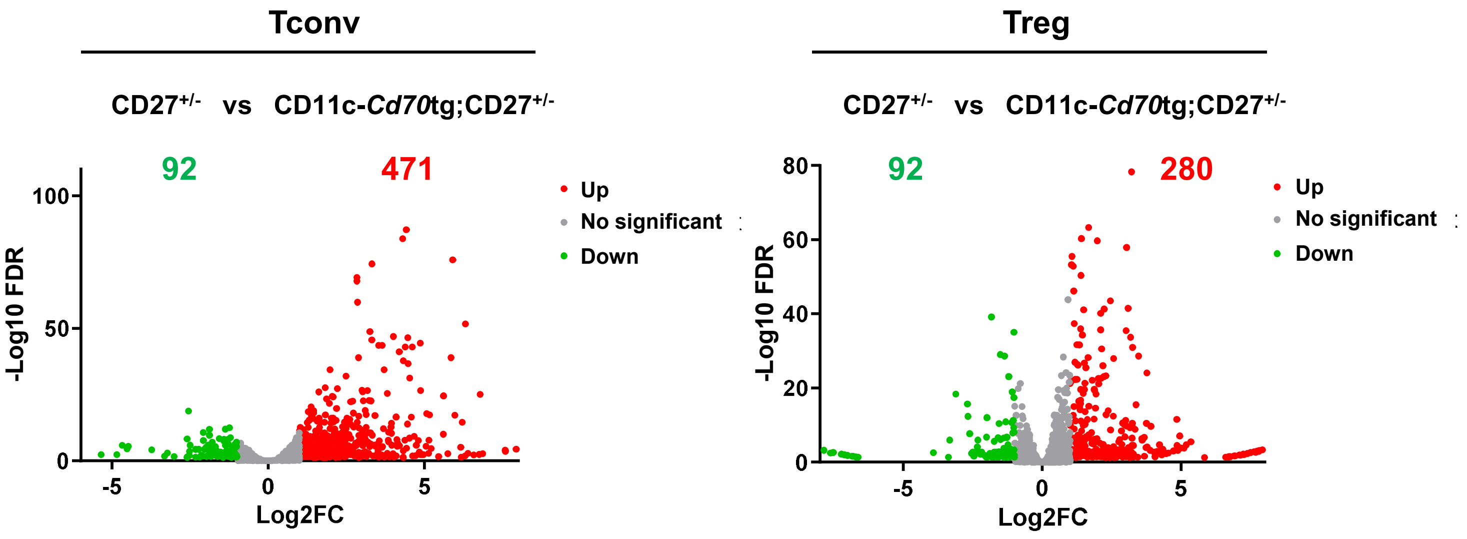

Supplemental Figure 6 : Volcano plot analysis of differential genes profile in CD27$^{+/-}$ and CD11c *Cd70*tg;CD27$^{+/-}$ mice; related to figure 6

Volcano Plot ; Tconvs : 563 were statistically differentiated ; 471 up and 92 down-regulated in Tconvs CD70tg. Tregs : 372 genes, 280 up and 92 down in Tregs CD70 tg

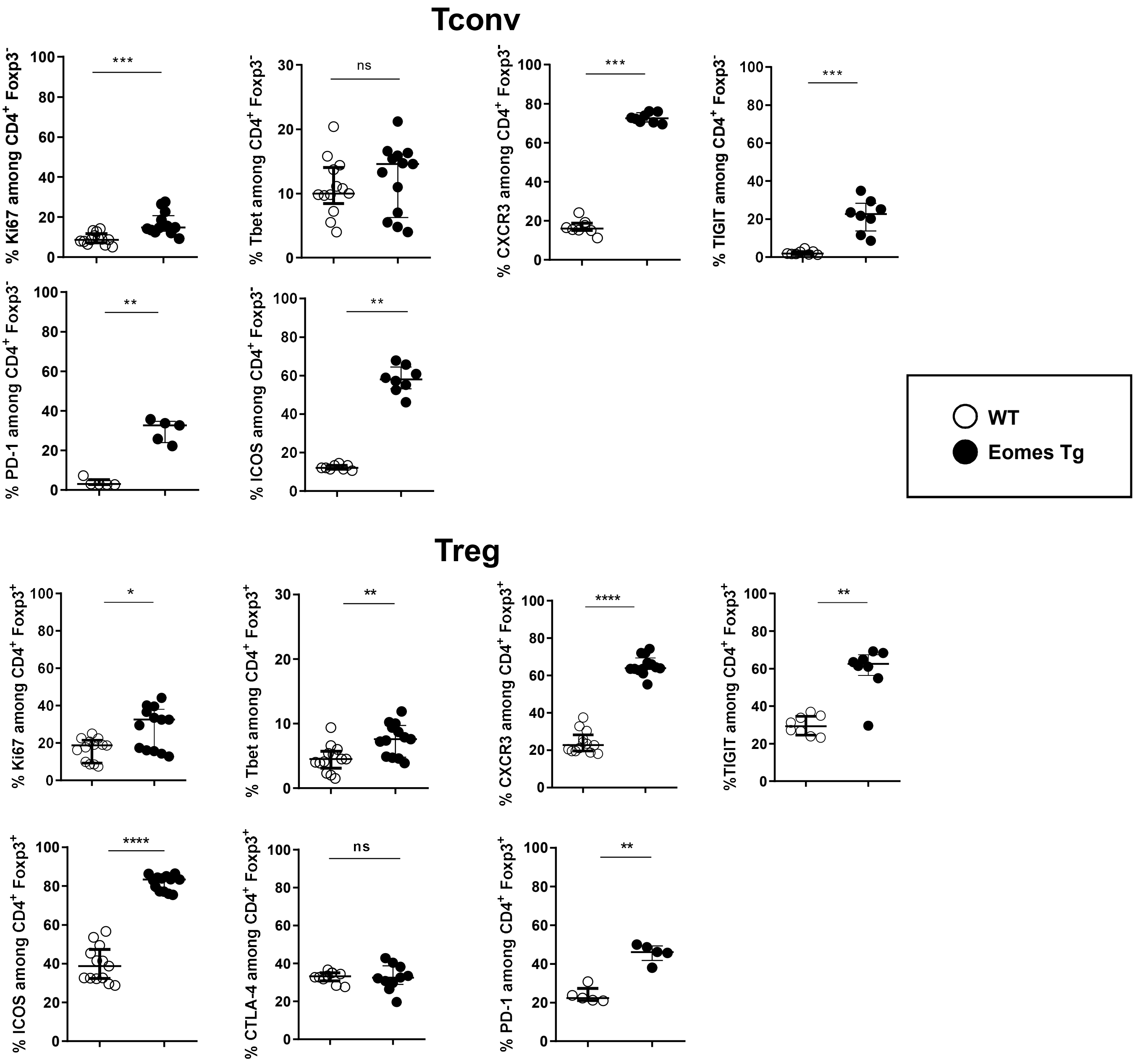

Supplemental Figure 7 : $Eomes^{tg}$ Tregs and Tconvs display Th1-like effector phenotype

Spleen cells from WT C57BL/6 and Eomes^{tg} mice were isolated and stained for proliferation marker Ki67, transcription factor T-bet, chemokine receptor CXCR3 and inhibitory receptors TIGIT, ICOS, CTLA-4 and PD-1 and analyzed by flow cytometry. Proportion of CD4$^+$ Tregs (upper panels) and Tconvs (lower panels) expressing the indicated marker. A non-parametric Mann-Whitney U test was used to determine statistical differences (*p<0.05; **p<0.01; ns, not significant). Bars represent mean with SEM of 5-13 individual mice. Data are representative of 3 independent experiments.

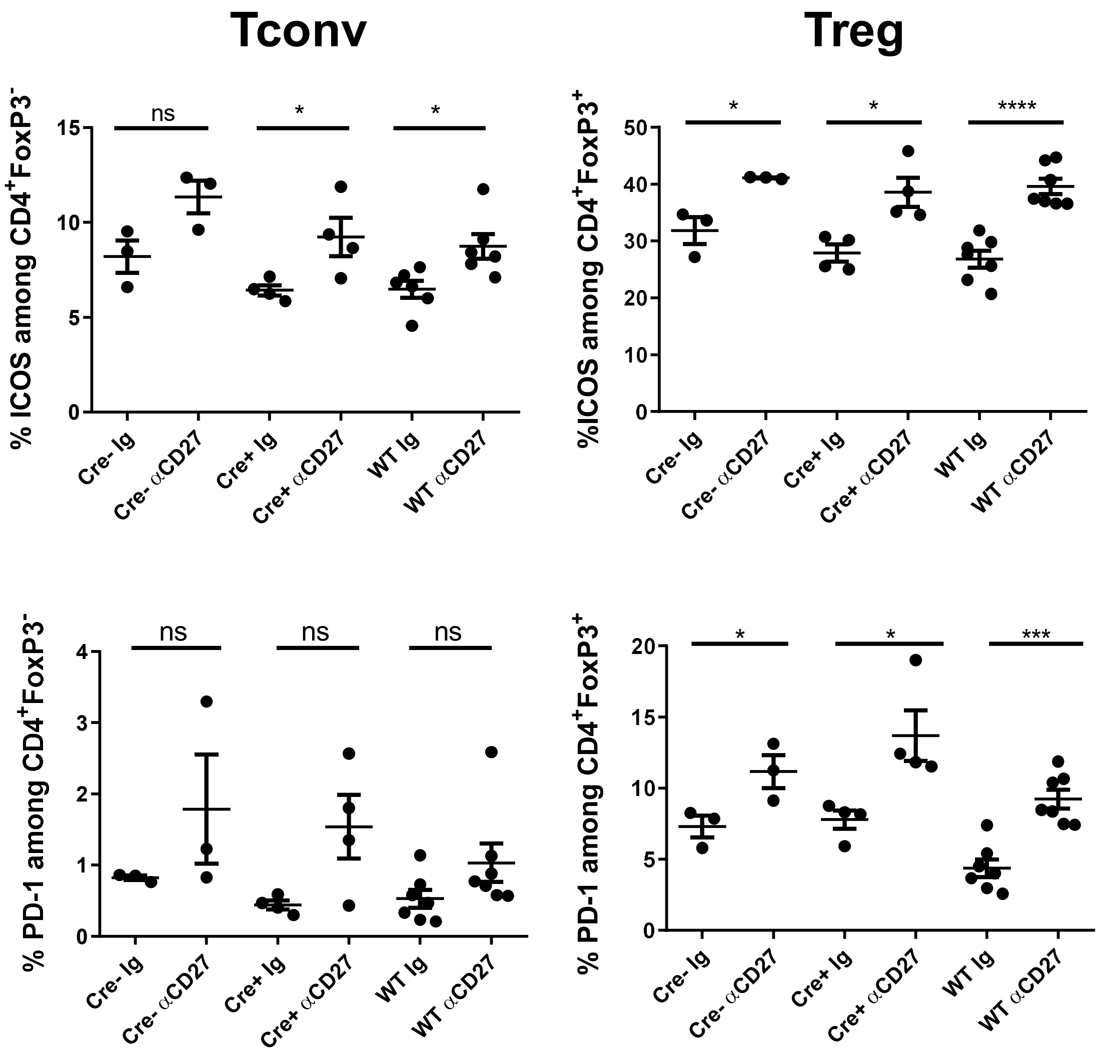

Supplemental Figure 8 : Eomes is dispensable for the CD27-induced activation of Th1-type Tregs

Mice with *lox*P-flanked *Eomes* alleles were crossed onto $CD4^{Cre}$ mice, giving rise to the $EOMES^{fl/fl}$ $CD4^{Cre}$ mouse strain harboring a selective deletion of *Eomes* in T lymphocytes. $EOMES^{fl/fl}$ $CD4^{Cre+}$, $EOMES^{fl/fl}$ $CD4^{Cre-}$ littermates and WT mice were injected i.p. with 100 µg agonistic anti-CD27 mAb or control Ig (at days 0 and 3) and spleen cells were analyzed *ex vivo* by flow cytometry at day 6.
Spleen cells were stained for CD4, Foxp3, ICOS and PD-1. Singlets were selected by gating events in the diagonal of FSC-H vs FSC-A plots. The data show the proportion of $CD4^+$ Tregs and Tconvs expressing inhibitory receptors (ICOS and PD-1). Data are from 1 experiment with 3-7 mice per group. Bars represent mean with SEM. Unpaired t-test was used to determine statistical differences (*p<0.05; ****p<0.0001; ns, not significant).
